# Gegen Qinlian decoction alleviates ulcerative colitis by remodeling the gut microbiota-bile acid axis and inhibiting NLRP3-mediated pyroptosis

**DOI:** 10.3389/fimmu.2026.1895206

**Published:** 2026-10-01

**Authors:** Hao Wang, Guosen Ou, Huachong Xu, Tangjuan Liu, Yawen Jiang, Shiqi Wang, Jialin Wu, Sha Wu, Li Deng, Lu Xu

**Affiliations:** 1 School of Traditional Chinese Medicine, Jinan University, Guangzhou, China; 2 Postdoctoral Station of Clinical Medicine, The First Affiliated Hospital of Jinan University, Guangzhou, China; 3 School of Basic Medicine and Public Health, Jinan University, Guangzhou, China; 4 Guangdong Provincial Key Laboratory of Traditional Chinese Medicine Informatization, Jinan University, Guangzhou, China; 5 Guangzhou Key Laboratory of Formula-Pattern of Traditional Chinese Medicine, School of Traditional Chinese Medicine, Jinan University, Guangzhou, China

**Keywords:** bile acids, Gegen Qinlian decoction, gut microbiota, pyroptosis, ulcerative colitis

## Abstract

**Background:**

Gegen Qinlian Decoction (GQD) is used to treat intestinal disorders, but its mechanism in ulcerative colitis (UC) induced by combined high-fat diet (HFD) and dextran sulfate sodium (DSS) exposure remains unclear.

**Methods:**

A UC mouse model was induced by HFD plus DSS. GQD constituents were identified by UPLC–MS/MS. Colonic pathology and barrier integrity were assessed by H&E and immunofluorescence. Gut microbiota and bile acids were analyzed by 16S rRNA gene sequencing and targeted metabolomics. Transcriptomics, ELISA, Western blotting, fecal microbiota transplantation (FMT), and molecular docking were used to investigate mechanisms. LPS-stimulated Caco-2 cells were treated with 20 or 50 μM DCA; cell viability, NLRP3 inflammasome-related gene expression, and ZO-1/Occludin immunofluorescence were evaluated.

**Results:**

GQD ameliorated gut dysbiosis, decreasing the Firmicutes/Bacteroidetes ratio, increasing *Akkermansia muciniphila*, and reducing *Clostridium perfringens*. It remodeled bile acid metabolism, decreasing primary bile acids (CA, TCA, and CDCA, etc.) and increasing secondary bile acids (DCA, 6,7-DKLCA and 3β-DCA, etc.). Transcriptomics indicated inhibition of NOD-like receptor signaling. GQD suppressed NLRP3 inflammasome activation and reduced ASC, pro-caspase-1, cleaved caspase-1, GSDMD-N, and IL-18. Docking prioritized DCA as a candidate NLRP3-interacting metabolite. In Caco-2 cells, DCA dose-dependently reduced LPS-induced NLRP3, ASC, GSDMD, IL1B, and IL18 mRNA expression and partially restored ZO-1 and Occludin signals without appreciable cytotoxicity. FMT supported the involvement of gut microbiota in GQD-mediated anti-inflammatory effects.

**Conclusion:**

GQD alleviates UC induced by combined HFD and DSS exposure by reshaping the gut microbiota and correcting bile acid dysregulation, thereby inhibiting NLRP3 inflammasome activation. This study highlights the gut microbiota–bile acid axis in GQD’s therapeutic effects.

## Graphical Abstract

**Figure:**
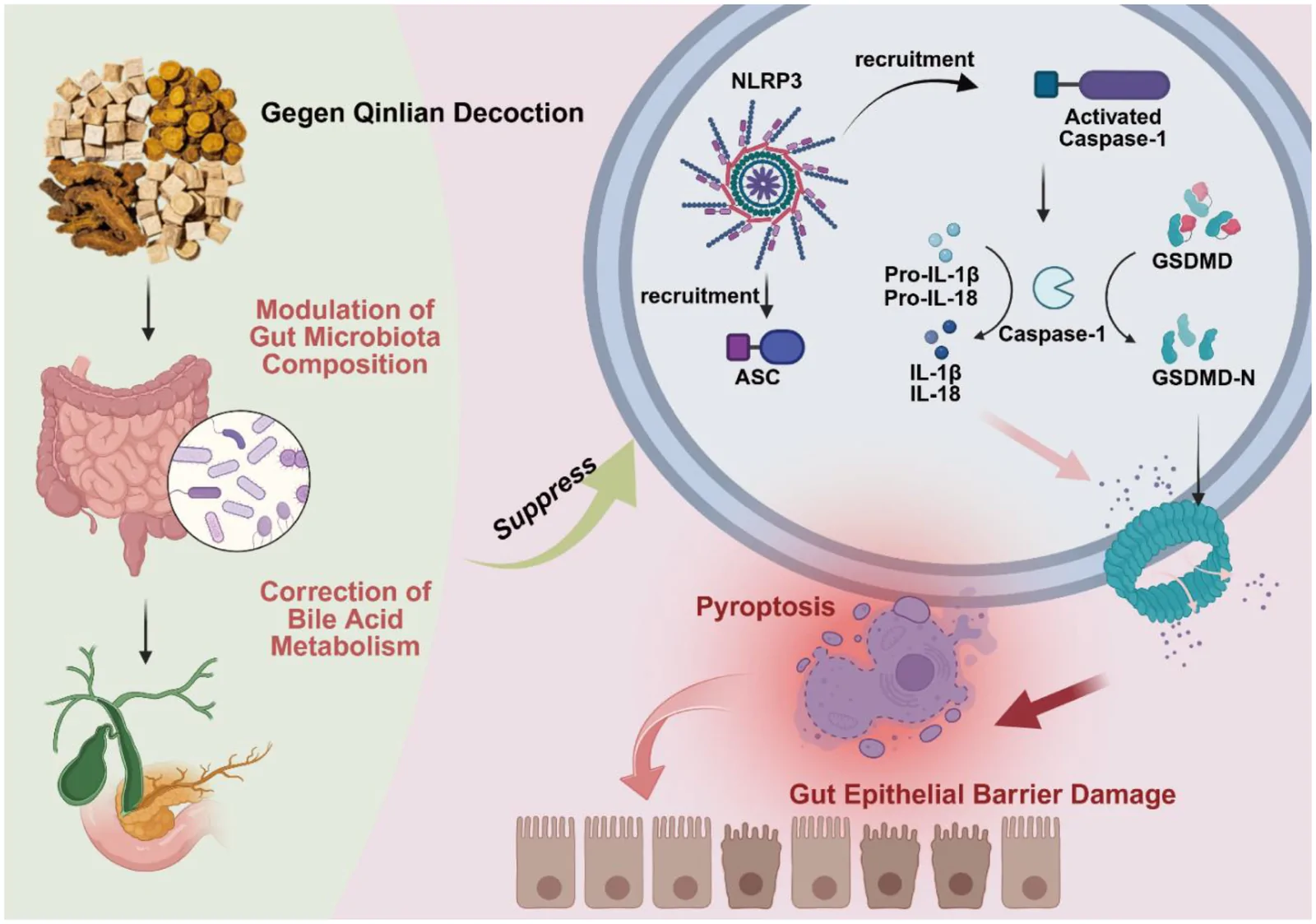

## 1 Introduction

Ulcerative colitis is a type of inflammatory bowel disease (IBD) characterized by chronic and recurrent inflammation of the colonic and rectal mucosa (1). Epidemiological studies indicate that since the onset of the 21st century, the incidence of UC has exhibited a continuous upward trend in emerging economies such as Asia, Africa, and Latin America (2). The prevalence of UC in China has also increased annually, becoming a significant public health issue (3). Currently, clinical management of UC primarily relies on 5-aminosalicylic acid (5-ASA) preparations, glucocorticoids, immunosuppressants, and biological agents (4). However, the single-target mechanisms of these drugs struggle to achieve systemic repair of the intestinal microenvironment and face notable application constraints in patients with underlying comorbidities. Consequently, exploring safe and effective alternative or adjuvant therapeutic strategies is of paramount importance.

Over the past half-century, alongside the global “Westernization” of dietary patterns, epidemiological and clinical observations have demonstrated that an HFD not only increases the risk of UC but also frequently triggers disease relapse or exacerbates clinical symptoms, suggesting its critical role in the pathogenesis and progression of UC (5). Previous research has demonstrated that an HFD exacerbates intestinal inflammatory responses by inducing gut dysbiosis and impairing the integrity of the intestinal mucosal barrier (6, 7). Microbial dysbiosis leads to significant alterations in the metabolic processes of intestinal bile acids (BAs); specifically, the decline in the abundance of bacteria possessing bile salt hydrolase (BSH) activity hinders the conversion of primary bile acids to secondary bile acids, resulting in the aberrant accumulation of primary bile acids.

As vital metabolites mediating the interaction between the microbiota and the host, the biotransformation of bile acids is intricately linked to the composition of the intestinal microbiome. Primary bile acids synthesized by hepatocytes are conjugated with taurine or glycine and secreted into the intestine, where gut microbiota-mediated deconjugation and metabolism convert the unabsorbed fraction into secondary bile acids (8). Accumulating clinical evidence has demonstrated that bile acid metabolic dysregulation is prevalent in UC patients, characterized by an increased proportion of primary bile acids and a decreased proportion of secondary bile acids (9, 10). Within this context, the aberrant accumulation of primary bile acids may not only directly impair the integrity of the intestinal epithelial barrier but also function as damage-associated molecular patterns (DAMPs) that activate the innate immune system, specifically triggering the NLRP3 inflammasome-mediated inflammatory cascade, thereby exacerbating intestinal inflammation (11). The NLRP3 inflammasome is a pivotal multiprotein signaling complex within the innate immune system; upon activation, it promotes the maturation and release of IL-1β and IL-18 via the cleavage of Caspase-1, and the sustained high expression of these pro-inflammatory cytokines is closely associated with immune imbalance and mucosal damage in colonic tissues (12).

Traditional Chinese Medicine has accumulated extensive experience in the prevention and treatment of chronic inflammatory diseases and has demonstrated unique advantages in the management of UC. Gegen Qinlian Decoction (GQD), originating from the *Shanghan Lun* (Treatise on Febrile Diseases), is composed of *Pueraria lobata* (Willd.) Ohwi, *Scutellaria baicalensis* Georgi, *Coptis chinensis* Franch., and *Glycyrrhiza uralensis* Fisch. Traditionally, GQD has been used to treat ‘Xie Li’ (dysentery-like syndrome) characterized by diarrhea, tenesmus, and bloody stools—symptoms that closely resemble the clinical presentation of active UC (13). Consistent with its traditional use, findings from clinical trials, quantitatively synthesized in a recent overview of systematic reviews, further confirmed that GQD combined with conventional medicine is more effective than conventional medicine alone in treating UC, as evidenced by improved response rates, reduced relapse rates, and better UCEIS scores (14). Recent studies suggest that GQD possesses anti-inflammatory properties, improves intestinal mucosal damage, and modulates the gut microecology; however, the specific mechanisms underlying its intervention in UC have not yet been fully elucidated (15).

In this study, we employed an HFD-primed, DSS-induced UC mouse model combined with 16S rRNA sequencing, targeted BA metabolomics, transcriptomics, and fecal microbiota transplantation to investigate the hypothesis that GQD reshapes the gut microbiota structure and BA profile, thereby suppressing NLRP3 inflammasome activation and pyroptosis, ultimately relieving intestinal inflammation and barrier damage.

## 2 Materials and methods

### 2.1 Reagents and materials

The constituent herbs of Gegen Qinlian Decoction—*Pueraria lobata* (Willd.) Ohwi (HX25K01), *Scutellaria baicalensis* Georgi (HX25L01), *Coptis chinensis* Franch. (HX25K01), and *Glycyrrhiza uralensis* Fisch. (HX25K01)—were all obtained from the First Affiliated Hospital of Jinan University (Guangzhou, China). Quantitative ELISA kits for mouse interleukin-18 (IL-18) (BYabscience, BY-EM220173) and mouse tumor necrosis factor-α (TNF-α) (BYabscience, BY-EM220852) were purchased from Nanjing BYabscience Biotechnology Co., Ltd. Also, Ampicillin (A800429), metronidazole (M813526), neomycin (N799581), and vancomycin (V871983) were purchased from Macklin (Shanghai, China). The high-fat diet (60% kcal from fat) was purchased from Research Diets, Inc.

### 2.2 Preparation of GQD

Based on the clinical dosage (16), the following raw herbs were weighed: *Pueraria lobata* (Willd.) Ohwi (15 g), *Scutellaria baicalensis* Georgi (9 g), *Coptis chinensis* Franch. (9 g), and *Glycyrrhiza uralensis* Fisch. (6 g). The herbal materials were macerated in a 10-fold volume (v/w) of distilled water for 30 min and subsequently decocted for 30 min. The resulting decoction was filtered, and the residue was re-extracted with an equal volume of distilled water. The two filtrates were combined and concentrated under reduced pressure at 70 °C to achieve the desired volume.

### 2.3 Identification of GQD by UPLC-MS/MS

The chemical composition of GQD was characterized by widely targeted metabolomics using ultra-performance liquid chromatography (UPLC) (Exion LC™ AD system, Sciex) coupled with tandem mass spectrometry (MS/MS). Chromatographic separation was performed on an Agilent SB-C18 column (1.8 μm, 2.1 mm × 100 mm). The mobile phases consisted of (A) ultrapure water containing 0.1% formic acid and (B) acetonitrile containing 0.1% formic acid. The gradient elution program was set as follows: 5% B at 0.00 min, linearly increased to 95% B within 9.00 min and maintained until 10.00 min, then decreased to 5% B within 1.10 min, and re-equilibrated at 5% B until 14.00 min. The flow rate was 0.35 mL/min, the column temperature was maintained at 40 °C, and the injection volume was 2 μL.

Mass spectrometric analysis was conducted with an electrospray ionization (ESI) source operated at a source temperature of 500 °C. The ion spray voltage (IS) was set at +5500 V in positive-ion mode and −4500 V in negative-ion mode. Ion source gas I (GSI), gas II (GSII), and curtain gas (CUR) were set at 50, 60, and 25 psi, respectively, with collision-activated dissociation (CAD) set to high. Data were acquired in multiple reaction monitoring (MRM) mode using nitrogen as the collision gas at medium pressure. Declustering potential (DP) and collision energy (CE) were optimized for each MRM ion pair, and the corresponding transitions were monitored according to scheduled elution time windows of the metabolites.

Metabolite identification was based on the Metware Database (MWDB) by matching secondary mass spectral fragments, excluding isotopic and adduct ion interferences, and confirming retention times. Quantification was achieved by extracting the chromatographic peak areas of each metabolite in MRM mode, followed by cross-sample normalization, thereby enabling high-throughput and high-sensitivity detection and quantification of the chemical constituents in GQD.

### 2.4 Animal models and GQD treatment

Experiment I: Male C57BL/6J mice (8 weeks old, 20 ± 2 g) were purchased from SiPeiFu Biotechnology Co., Ltd. (License No.: SCXK2022-0063). Mice were randomly assigned to five groups: normal control (NC), DSS model (Model), low-dose GQD (GQD-L), high-dose GQD (GQD-H), and 5-aminosalicylic acid (5-ASA). The NC group was fed a standard maintenance diet, while the other groups were fed an HFD for 2 weeks. Subsequently, UC was induced in all mice except the NC group using 3% DSS. Simultaneously, the NC and Model groups received an equal volume of normal saline, while the intervention groups were administered treatments once daily via oral gavage for 7 consecutive days. Dosages, converted based on human-to-mouse body surface area, were as follows: GQD-L (6.9 g/kg), GQD-H (13.7 g/kg), and 5-ASA (50 mg/kg). All mice had ad libitum access to food and water and were maintained in a 12-h light/dark cycle. All experimental procedures were approved by the Animal Ethics Committee of Jinan University (Approval No.: 20250704-05).

Experiment II: Twelve mice were randomly divided into two groups and initially treated with a broad-spectrum antibiotic (ABX) cocktail to deplete the gut microbiota. Recipient mice were pre-treated with an antibiotic cocktail (ABX) (ampicillin 1 g/L, metronidazole 1 g/L, neomycin 1 g/L, and vancomycin 0.5 g/L) in their drinking water for 14 days, while being fed an HFD. Following ABX treatment, mice were switched to regular drinking water for 2 days to wash out residual antibiotics. Subsequently, UC was induced in both groups using 3% DSS for one week. Fecal microbiota transplantation (FMT) was initiated concurrently with 3% DSS administration. Fresh feces (200 mg) were collected daily from the cages of the Model and GQD-H groups and immediately suspended in 5 mL of normal saline. The suspension was vortexed for 3 min and allowed to stand for 2 min; the resulting supernatant (200 µL) was then transplanted into recipient mice once daily for 7 days. Briefly, following microbiota depletion and DSS induction, mice in the FMT-Model and FMT-GQD groups were administered fecal suspensions from the Model and GQD-H donor groups, respectively, for one week.

### 2.5 Histopathological examination

#### 2.5.1 Hematoxylin and eosin staining

Colonic tissues were fixed in 4% paraformaldehyde, trimmed, and processed according to standard protocols, including washing, dehydration, clearing, paraffin embedding, and sectioning. Sections were deparaffinized, rehydrated, and stained with H&E. Histological evaluation was performed using an optical microscope.

#### 2.5.2 Immunofluorescence staining

Paraffin sections were baked at 65 °C for 2 h, deparaffinized in xylene, and rehydrated through a graded ethanol series. Antigen retrieval was performed using EDTA buffer (pH 9.0) at 95 °C for 20 min. Sections were subsequently blocked with 3% hydrogen peroxide and goat serum. Colonic sections were incubated with anti-ZO-1 (Servicebio, GB111401, 1:1000) and anti-Occludin (Servicebio, GB151981, 1:1000) antibodies. Following washing, Tyramide Signal Amplification (TSA) was performed using TYR-570 (for ZO-1) and TYR-520 (for Occludin). Nuclei were counterstained with DAPI, and images were captured using a confocal microscope.

### 2.6 Western blot analysis

Total protein was extracted from colon tissues using ice-cold RIPA lysis buffer (Beyotime, P0013G) supplemented with 1 mM protease and phosphatase inhibitors. After homogenization, the lysates were incubated on ice for 30 min, followed by centrifugation at 12,000 rpm for 10 min (at 4 °C) to collect the supernatants. Protein concentrations were determined using a BCA Protein Assay Kit (Solarbio, Beijing, China). Equal amounts of protein were resolved by 10% SDS-PAGE and electrotransferred onto PVDF membranes. After blocking with 5% non-fat dry milk for 2 h at room temperature, the membranes were incubated with primary antibodies (detailed in **Supplementary Table 1**) overnight at 4 °C. Subsequently, the membranes were incubated with HRP-conjugated secondary antibodies for 2 h at room temperature. Protein bands were visualized using enhanced chemiluminescence (ECL) reagents (Meilunbio, MA086-2), and their intensities were quantified via densitometric analysis using ImageJ software, with β-actin serving as the internal loading control.

### 2.7 Enzyme-linked immunosorbent assay

The levels of IL-18 and TNF-α in murine colonic tissues were quantified using commercially available ELISA kits (BYabscience Biotechnology Co., Ltd.) in strict accordance with the manufacturer’s protocols. Briefly, 50 μL of each sample was added to the designated wells, and the optical density (OD) was measured at 450 nm using a microplate reader. Cytokine concentrations were calculated by interpolating from a standard curve generated via ELISACalc software.

### 2.8 16S rRNA gene sequencing and microbiota analysis

Total genomic DNA was extracted from microbial samples using the cetyltrimethylammonium bromide (CTAB) method, with DNA purity and concentration monitored via agarose gel electrophoresis. The V4 hypervariable region of the 16S rRNA gene was amplified using specific barcoded primers (515F and 806R) and Phusion^®^ High-Fidelity PCR Master Mix with GC Buffer (New England Biolabs, USA). The resulting PCR products were visualized on 2% agarose gels, purified using magnetic beads, and pooled in equimolar ratios. Target fragments were subsequently retrieved using a gel extraction kit. Sequencing libraries were constructed using the TruSeq^®^ DNA PCR-Free Sample Preparation Kit, validated via Qubit and quantitative PCR (qPCR), and sequenced on the Illumina NovaSeq 6000 platform in paired-end mode. Sequences were clustered into operational taxonomic units (OTUs) using the Uparse algorithm (USEARCH v7) based on a ≥ 97% similarity threshold. Multiple sequence alignment was performed using MAFFT (v7.520) to infer phylogenetic relationships. To account for differences in sequencing depth, all samples were rarefied to the minimum library size. Alpha and Beta diversity indices were subsequently calculated based on the normalized data. Sequencing and bioinformatic analysis were performed by Metware Biotechnology Co., Ltd. (Wuhan, China).

### 2.9 Targeted bile acid metabolomics

Targeted quantification of bile acids (BAs) in murine cecal contents was performed using liquid chromatography-tandem mass spectrometry (LC-MS/MS). Briefly, 20 mg of solid sample was accurately weighed and supplemented with steel beads and 10 μL of internal standard working solution (1 μg/mL). BAs were extracted via homogenization in 200 μL of 20% methanol/acetonitrile (v/v). The mixture was vortexed at 2500 rpm for 10 min, incubated at -20 °C for 10 min, and then centrifuged at 12,000 rpm for 10 min at 4 °C. The resulting supernatant was collected for LC-MS/MS analysis. Chromatographic separation was achieved using a Waters ACQUITY UPLC HSS T3 C18 column (1.8 μm, 100 mm × 2.1 mm) maintained at 40 °C. Mobile phase A consisted of ultrapure water containing 0.01% acetic acid and 5 mmol/L ammonium acetate, while mobile phase B was acetonitrile containing 0.01% acetic acid. The gradient elution program was optimized as follows: 0 min, 95% A; 0.5 min, 60% A; 4.5 min, 50% A; 7.5 min, 25% A; 10 min, 5% A; and 12 min, 95% A. The flow rate was set to 0.35 mL/min with an injection volume of 1 μL. Mass spectrometry was conducted on a SCIEX QTRAP 6500+ system equipped with an electrospray ionization (ESI) source operating in negative ion mode. The ion source temperature was 550 °C, the ion spray voltage was -4500 V, and the curtain gas pressure was 35 psi. Sixty-five bile acids were quantified using multiple reaction monitoring (MRM), with declustering potential (DP) and collision energy (CE) optimized for each transition. Metabolite identification was based on a proprietary MWDB standard database, and quantification was performed by integrating peak areas in MRM mode and interpolating from corresponding standard curves. Data were processed using Analyst (v1.6.3) and MultiQuant (v3.0.3). Data quality was assessed via total ion chromatogram (TIC) overlap, quality control (QC) correlation, and coefficient of variation (CV) analysis. Principal component analysis (PCA) and orthogonal projections to latent structures-discriminant analysis (OPLS-DA) were employed to identify metabolic shifts. Differential metabolites were screened based on a combination of fold-change (FC) with |log2FC| > 1, variable importance in projection (VIP) > 1, and P < 0.05, followed by functional annotation and enrichment analysis via KEGG and HMDB databases. The metabolomics analysis was performed by Metware Biotechnology Co., Ltd. (Wuhan, China).

### 2.10 RNA sequencing and transcriptomic analysis

Total RNA was extracted using TRIzol reagent (Invitrogen, USA), and its concentration and integrity were rigorously assessed using a Qubit fluorometer (Thermo Fisher Scientific) and a Qsep400 bioanalyzer (BiOptic), respectively. For library construction, polyadenylated mRNA was enriched from total RNA using Oligo(dT) magnetic beads and subsequently fragmented. First-strand cDNA was synthesized using random hexamer primers, followed by second-strand synthesis. For strand-specific libraries, dUTP was incorporated in place of dTTP during second-strand synthesis; this ensures strand orientation, as uracil-containing templates are not amplified by high-fidelity DNA polymerases. Following end repair, A-tailing, adapter ligation, and size selection, the fragments were amplified via PCR. Final libraries were circularized to form single-stranded DNA and transformed into DNA nanoballs (DNBs) for high-throughput sequencing on the BGI platforms.

Raw sequencing data were processed using fastp (v0.23.2) to remove adapters and low-quality reads (defined as N content > 10% or > 50% of bases with Q ≤ 20). Clean reads were aligned to the reference genome using HISAT2 (v2.2.1). Gene expression levels were quantified using featureCounts (v2.0.3) and normalized as Fragments Per Kilobase of transcript per Million mapped reads (FPKM). Differential expression analysis was performed using DESeq2 (v1.38.3) for samples with biological replicates and edgeR (v3.40.2) for those without, with significance defined as |log_2_FoldChange|≥1 and (FDR) < 0.05. The cDNA libraries were sequenced on the BGI platform by Metware Biotechnology Co., Ltd. (Wuhan, China).

### 2.11 Cell-based experiments

#### 2.11.1 Cell culture

The human colorectal adenocarcinoma cell line Caco-2 (JCM-H1040; Hangzhou Jcellm Biological Technology Co., Ltd.) was cultured in Eagle’s Minimum Essential Medium (EMEM; G4650-500ML, Servicebio) supplemented with 20% fetal bovine serum (FBS) and 1% penicillin–streptomycin. Cells were maintained at 37 °C in a humidified incubator containing 5% CO_2_. When cell confluence reached 80%–90%, the cells were passaged using 0.25% trypsin–EDTA. Cells with comparable passage numbers and confirmed to be negative for mycoplasma contamination were used in the experiments.

#### 2.11.2 Assessment of DCA cytotoxicity using the CCK-8 assay

Caco-2 cells were seeded into clear, flat-bottom 96-well plates at a density of 5.0 × 10³ cells/well in 100 μL of complete culture medium per well and cultured for 24 h. In the initial experiment, a standard curve covering seeding densities of 2,000–20,000 cells/well was established to confirm that the OD_450_ values remained within the linear range at the assay endpoint. DCA (D4297, Sigma-Aldrich) was dissolved in DMSO to prepare a 200 mmol/L stock solution (78.5 mg/mL), from which 1,000× working solutions at concentrations of 20, 50, 100, and 200 mmol/L were prepared in DMSO. Each working solution was added to the culture medium at 1 μL/mL to achieve final DCA concentrations of 20, 50, 100, or 200 μmol/L. The DMSO vehicle control received an equivalent volume of DMSO, and the final DMSO concentration was maintained at 0.1% (v/v) in the vehicle control and all DCA-treated groups. An untreated control (NC) without DMSO or DCA was included. Three technical replicate wells were included for each concentration, together with cell-free blank wells. After the culture medium was replaced with medium containing the indicated concentration of DCA or vehicle, the cells were cultured for an additional 24 h. CytoDet CCK-8 reagent (20 μL; AG51015, Accurate Biology) was added to each well, followed by incubation at 37 °C for 2 h in the dark. After gentle mixing and confirmation that no air bubbles were present, absorbance was measured at 450 nm. Cell viability was normalized to the untreated NC group, defined as 100%. Five independent experiments were performed, and each plotted point represents one independent experiment. Based on the preliminary CCK-8 screening results, 50 μM DCA was selected as the high concentration (DCA-H), and 20 μM was selected as the low concentration (DCA-L) for subsequent experiments.

#### 2.11.3 LPS stimulation and DCA intervention

To evaluate the effect of DCA on lipopolysaccharide (LPS)-induced activation of the NLRP3 inflammasome, Caco-2 cells were seeded into 6-well plates at a density of 3.0 × 10^5^ cells/well in 2 mL of complete culture medium per well and cultured for 24 h. The medium was then replaced with fresh complete culture medium, and the cells were treated with LPS and/or DCA (D4297, Sigma-Aldrich) for 24 h according to the experimental grouping. LPS and DCA were added simultaneously to the combination groups.

Four groups were included in the RT-qPCR and immunofluorescence experiments: NC, LPS, LPS plus DCA-L (20 μM), and LPS plus DCA-H (50 μM). All groups contained 0.1% DMSO (v/v), and NC cells received vehicle without LPS or DCA. LPS (L4391, Sigma-Aldrich) was purchased, prepared in complete culture medium and applied at a final concentration of 10 μg/mL. After 24 h of treatment, the cells were collected for RNA extraction.

For the cell-viability experiment, an additional DCA-H-alone group was included, resulting in five groups: NC, LPS, LPS plus DCA-L, LPS plus DCA-H, and DCA-H alone. All groups contained 0.1% DMSO and were treated for 24 h. Cell viability was measured using the CCK-8 assay described above and normalized to the vehicle-containing NC group, defined as 100%.

### 2.12 RT-qPCR

Total RNA was extracted separately from the cells in each group using the SteadyPure RNA Extraction Kit (AG21024, Accurate Biology) according to the manufacturer’s instructions, without pooling the samples. RNA concentration and purity were determined using a NanoDrop microvolume spectrophotometer (Thermo Fisher Scientific). Only RNA samples with A_260_/A_280_ ratios of 1.8–2.1 and A_260_/A_230_ ratios of ≥1.8 were used. For each sample, 500 ng of RNA was subjected to reverse transcription in a total reaction volume of 20 μL using the Evo M-MLV RT Mix Kit with gDNA Clean for qPCR Ver. 2 (AG11728, Accurate Biology). The reaction program consisted of genomic DNA removal at 42 °C for 2 min, reverse transcription at 37 °C for 15 min, and reaction termination at 85 °C for 5 s.

The qPCR primer sequences were obtained from PrimerBank or selected from sequences validated in previously published studies and were synthesized by Biosune Biotechnology (Shanghai) Co., Ltd.

qPCR was performed using the SYBR Green Pro Taq HS qPCR Kit (AG11701, Accurate Biology) on a Bio-Rad CFX96 Real-Time PCR System. The amplification program consisted of an initial step at 95 °C for 30 s, followed by 40 cycles at 95 °C for 5 s and 60 °C for 30 s. Melt-curve analysis was subsequently performed. Each cDNA sample was analyzed in three technical replicate wells. The target genes examined were NLRP3, ASC, GSDMD, IL1B, and IL18, with GAPDH included as a reference gene.

### 2.13 Verification of target bacterial abundance and colonization consistency following FMT

Fresh fecal samples were collected from donor mice in the Model and GQD groups. Fecal suspensions were prepared and transplanted into the intestinal tract of recipient mice via oral gavage. Following colonization, fecal samples were collected from the recipient mice, and total bacterial DNA was extracted using a fecal DNA extraction kit. A 20 μL reaction mixture was prepared using the SYBR Green Pro Taq HS qPCR Kit (AG11701), with a final primer concentration of 0.2 μmol/L and 2 μL of template DNA. The reference amplification program consisted of an initial step at 95 °C for 30 s, followed by 40 cycles at 95 °C for 5 s and 60 °C for 30 s. Melt-curve analysis was subsequently performed. Each DNA sample was analyzed in three technical replicate wells.

### 2.14 Cellular immunofluorescence staining

Caco-2 cells were seeded at a density of 1.0 × 10^5^ cells/well in 24-well plates containing sterile 12-mm round coverslips. The cells were cultured for 48–72 h until they reached ≥90% confluence and formed a continuous monolayer, after which they were treated as described above. Following treatment, the cells were washed with PBS, fixed with 4% neutral paraformaldehyde for 15 min at room temperature, permeabilized with 0.1% Triton X-100 for 5 min at room temperature, and blocked with 5% normal goat serum for 1 h at room temperature.

A rabbit anti-ZO-1 polyclonal antibody (AF5145, Affinity Biosciences; 1:200) and a mouse anti-Occludin monoclonal antibody (BF8330, Affinity Biosciences; 1:200) were combined to prepare the primary antibody working solution. The samples were incubated with the primary antibody mixture overnight at 4 °C in a humidified chamber protected from light. After washing with PBS the following day, the samples were incubated with a secondary antibody mixture containing Cy3-conjugated goat anti-rabbit IgG (GB21303, Servicebio; 1:400) and Alexa Fluor 488-conjugated goat anti-mouse IgG (GB25301, Servicebio; 1:400) for 1 h at room temperature in the dark. After further washing with PBS, the nuclei were counterstained with ready-to-use DAPI (G1012-10ML, Servicebio) for 8–10 min at room temperature in the dark. The coverslips were then mounted using antifade mounting medium (G1401, Servicebio).

Images were acquired using a laser-scanning confocal microscope in the 405 nm (DAPI), 488 nm (Occludin), and 561 nm (ZO-1) channels. The laser power, gain, and exposure time for each channel were established in preliminary experiments using the absence of signal saturation in the group with the highest fluorescence intensity as the criterion and were subsequently kept constant throughout the experiment. Five non-overlapping fields of view were acquired from each coverslip.

### 2.15 Molecular docking

Molecular docking was performed using chain A of the NLRP3 structure (PDB ID: 7PZC) as the receptor. Candidate ligands were limited to secondary bile acids and their derivatives identified by LEfSe analysis based on the preceding gut microbiota analysis. The 8GI-binding pocket was defined as the docking region, with a grid center at x = 192.852, y = 204.720, and z = 119.662 and box dimensions of 24.000 × 24.000 × 24.000 Å.

Three-dimensional ligand conformations were generated using RDKit with ETKDGv3 embedding and were energy-minimized using MMFF94 or UFF. Ligand PDBQT files were prepared using Meeko. Docking was performed using AutoDock Vina 1.2.x with exhaustiveness = 32, num_modes = 20, energy_range = 5, cpu = 4, and seed = 20260701.

For each ligand, the lowest Vina affinity value among the generated modes was recorded as the docking score. Hydrogen bonds were analyzed independently using the Baker–Hubbard method (H···A ≤ 2.5 Å and D–H···A angle ≥ 120°) and a BINANA-like method (D···A ≤ 4.0 Å and D–H···A angle ≥ 140°). The maximum hydrogen-bond counts obtained by the two methods were reported separately and were not summed. The larger value was used as the primary ranking criterion, while the Vina docking score was used as a secondary criterion. The lowest Vina score and maximum hydrogen-bond count could originate from different docking modes and were therefore summarized across multiple docking poses.

### 2.16 Statistical analysis

All statistical analyses were performed using GraphPad Prism version 9.5. All data were subjected to normality tests prior to statistical analysis. Data following a normal distribution are expressed as the mean ± standard deviation (SD). For comparisons between two groups, an independent samples t-test was used for normally distributed data, while the Mann-Whitney U test was employed for non-normally distributed data. For comparisons among three or more groups, one-way analysis of variance (ANOVA) followed by LSD *post-hoc* multiple comparisons was used for normally distributed data. A p-value <0.05 was considered statistically significant (ns: *P*>0.05; *P < 0.05; **P < 0.01; ***P < 0.001; ****P < 0.0001).

## 3 Results

### 3.1 Qualitative analysis of the components of Gegen Qinlian decoction

Ultra-performance liquid chromatography-tandem mass spectrometry (UPLC-MS/MS) was employed to identify the chemical constituents of GQD (**Figure 1**). The acquired data were cross-referenced against the MetWare Database (MWDB) to annotate the detected compounds. A total of 2,582 unique metabolites (**Supplementary Table 2**) were identified. Additionally, chromatographic fingerprinting and comparative analyses were performed on 11 quality control markers or key bioactive compounds derived from the herbal components of GQD (**Table 1**).

**Figure 1:**
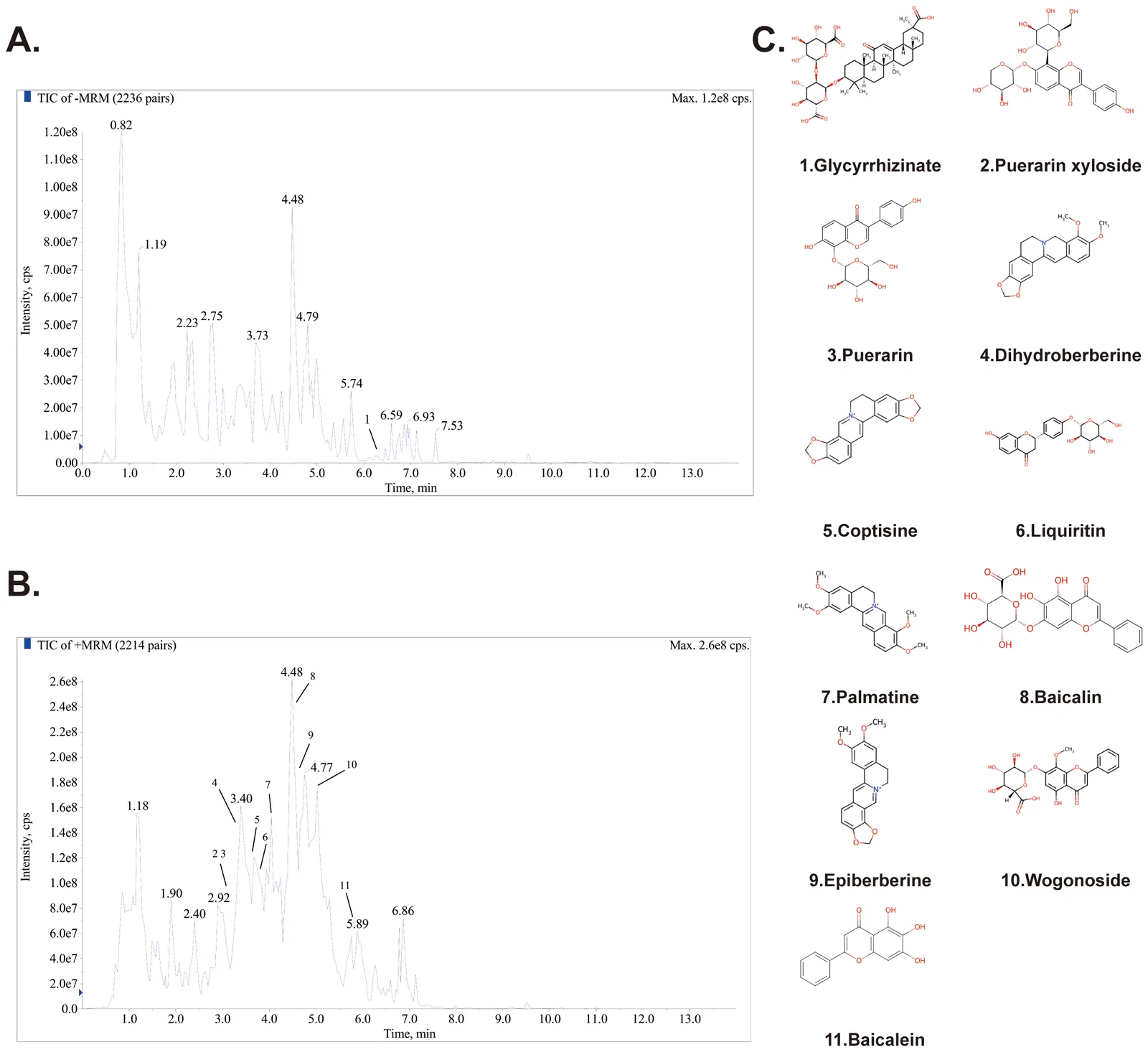
Qualitative analysis of GQD constituents based on UPLC/MS/MS and MWDB. **(A)** negative ion mode; **(B)** positive ion mode; **(C)** Chemical structures of the main components of GQD.

**Table 1:** Eleven characteristic compounds identified in GQD.

Compounds	Formula	Molecular weight (Da)	Ionization model	CAS	Label
Glycyrrhizinate	C42H62O16	822.4038	[M-H]-	1405-86-3	1
Puerarin xyloside	C26H28O13	548.153	[M+H]+	–	2
Puerarin	C21H20O9	416.1107	[M+H]+	3681-99-0	3
Dihydroberberine	C20H19NO4	337.1314	[M+H]+	483-15-8	4
Coptisine	C19H13NO4	319.0845	[M+H]+	3486-66-6	5
Liquiritin	C21H22O9	418.1264	[M+H]+	551-15-5	6
Palmatine	C21H22NO4+	352.1543	[M]+	3486-67-7	7
Baicalin	C21H18O11	446.0849	[M+H]+	21967-41-9	8
Epiberberine	C20H18NO4+	336.123	[M]+	6873-09-2	9
Wogonin-7-O-Glucuronide (Wogonoside)	C22H20O11	460.1006	[M+H]+	51059-44-0	10
Baicalein	C15H10O5	270.0528	[M+H]+	491-67-8	11

### 3.2 GQD ameliorates disease symptoms in mice with ulcerative colitis

Initially, Experiment I was conducted. Following two weeks of ad libitum HFD consumption, UC was induced using DSS in all groups except the NC group (**Figure 2A**). From day 3 to day 7 post-induction, all groups except the NC group exhibited a progressive decline in body weight, with the Model group showing the most pronounced reduction. Treatment with GQD-L, GQD-H, and 5-ASA significantly ameliorated the weight loss trend starting from day 5 (**Figure 2B**). Similar results were observed for the Disease Activity Index (DAI), where both high and low doses of GQD effectively reduced disease severity (**Figure 2C**). Morphologically, colonic tissues in the Model group were characterized by extensive hyperemia, edema, and significant shortening of colon length; however, intervention with GQD (both low and high doses) and 5-ASA improved these pathological manifestations to varying degrees, with high-dose GQD showing efficacy comparable to 5-ASA (**Figure 2D**). H&E staining revealed massive inflammatory cell infiltration, discontinuous epithelial structures, and a reduction in goblet cells in the colonic mucosa of the Model group, all of which were significantly attenuated following GQD treatment (**Figure 2E**). Immunofluorescence staining of intestinal barrier-related proteins showed that the expression of tight junction proteins ZO-1 and Occludin was decreased after DSS induction, while GQD treatment successfully upregulated their expression (**Figure 2F**). These findings demonstrate that GQD exerts a clear therapeutic effect on UC in an HFD-preconditioned DSS mouse model in mice, and the underlying mechanism may be closely related to the protection of intestinal barrier integrity.

**Figure 2:**
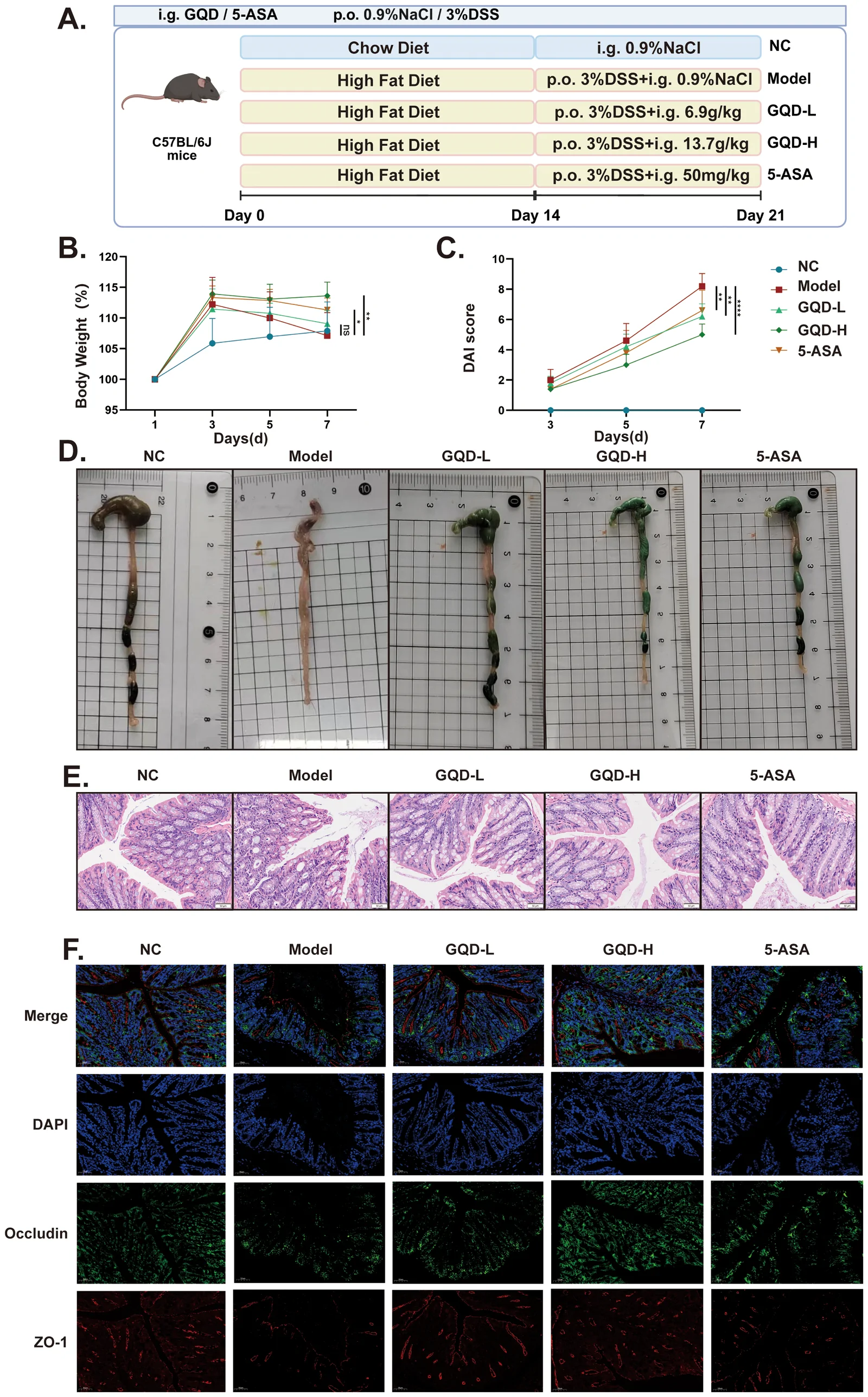
GQD ameliorates disease symptoms in mice with ulcerative colitis. **(A)** Schematic diagram of Experiment I; **(B)** Body weight changes in mice following DSS administration; **(C)** Disease Activity Index (DAI) score; **(D)** Representative colonic morphology; **(E)** H&E staining of colonic histological sections; **(F)** Immunofluorescence staining of ZO-1 and Occludin in the colon (ns, not significant; *P < 0.05; **P < 0.01; ***P < 0.001; oral gavage, i.g.; oral administration, p.o. n=5).

### 3.3 GQD modulates the gut microbiota in mice with ulcerative colitis

To investigate whether the therapeutic effects of GQD on DSS-induced UC are associated with the gut microbiota, fecal samples were collected from the NC, Model, and GQD-H groups for 16S rRNA gene amplicon sequencing. Principal Coordinates Analysis (PCoA) revealed a distinct separation between the groups, confirming significant differences in the composition of the gut microbiota (**Figure 3A**). Furthermore, Similarity Percentage (SIMPER) analysis was employed to identify the driver species contributing most significantly to the microbial differences between the Model and GQD groups. These primarily included *Akkermansia muciniphila*, *Bacteroides sartorii*, and *Escherichia coli*, which exhibited marked differences in relative abundance between the two groups (**Figure 3B**). Bar plots of relative abundance at the phylum, genus, and species levels illustrated the variations in microbial distribution and abundance across the groups. Compared with the Model group, GQD intervention led to a reduction in Firmicutes and an increase in Bacteroidota at the phylum level, resulting in a decreased Firmicutes/Bacteroidetes (F/B) ratio. At the genus level, the abundance of beneficial bacteria, such as *Akkermansia* and *Lactobacillus*, significantly increased, while the opportunistic pathogen Parasutterella decreased. At the species level, beneficial taxa including *Akkermansia muciniphila* and *Lactobacillus johnsonii* were significantly enriched (**Figures 3C–E**). LEfSe analysis showed that, compared to the NC group, the opportunistic pathogen *Clostridium perfringens* was markedly increased in the Model group, while the beneficial *Lactobacillus murinus* was significantly depleted. Compared to the Model group, the enrichment of *Akkermansia muciniphila* was most prominent following GQD intervention, accompanied by a significant reduction in *Clostridium perfringens* (**Figure 3F**). Notably, *Akkermansia muciniphila* and Lactobacillus murinus were consistently identified as highly differentially enriched species across multiple analyses, suggesting their pivotal roles in the GQD-mediated modulation of gut microbiota structure. These results indicate that the pathogenesis of DSS-induced UC is closely linked to gut dysbiosis, and GQD effectively ameliorates microbial imbalance and promotes the restoration of intestinal homeostasis by modulating the microbiota structure.

**Figure 3:**
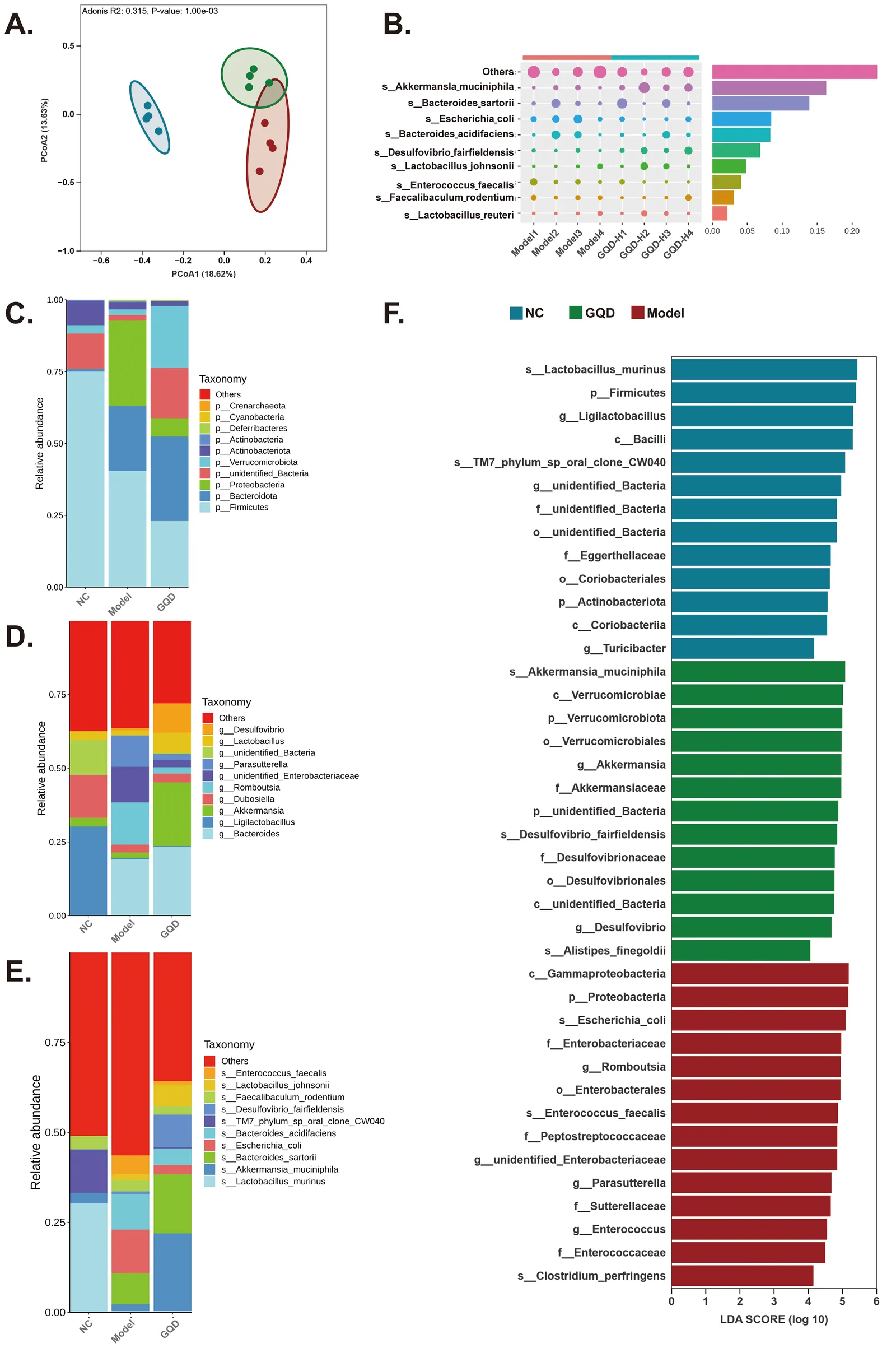
GQD modulates the gut microbiota in mice with ulcerative colitis. **(A)** Principal Coordinates Analysis (PCoA) of the microbial communities; **(B)** SIMPER analysis identifying the contribution of microbial species to the differences between groups; **(C–E)** Stacked bar plots representing the relative abundance of the gut microbiota at the phylum, genus, and species levels across the NC, Model, and GQD groups; **(F)** Linear Discriminant Analysis Effect Size (LEfSe) analysis of microbial taxa (n=4).

### 3.4 GQD modulates bile acid metabolism in the DSS-induced UC mouse model

Given the close association between the significantly altered microbiota and bile acid metabolism (17, 18) following GQD intervention, we further employed targeted metabolomics to systematically evaluate the regulatory effects of GQD on the intestinal bile acid profile of UC mice. Principal Component Analysis (PCA) showed a distinct separation in the bile acid metabolic profiles between the GQD and Model groups (**Figure 4A**). The VIP plot and the bar plots of differential metabolites from the OPLS-DA model further revealed significant differences in the relative levels of various bile acids between the two groups (**Figures 4B, C**). Further analysis of the most significantly altered bile acids showed an aberrant accumulation of multiple primary bile acids (including CA, CDCA, TCA, Tω-MCA, Tβ-MCA, and TCA-3S) in the intestines of the Model group. Conversely, GQD treatment significantly downregulated the levels of these primary bile acids. Meanwhile, the levels of certain secondary bile acids (such as 6,7-DKLCA and 3β-DCA) were increased to varying degrees in the GQD group compared to the Model group (**Figure 4D**). To elucidate the potential link between gut microbiota and bile acid metabolism, Spearman correlation analysis (*P* < 0.05) was performed between the 16 most significantly differential species identified via 16S rRNA sequencing and the differential bile acid metabolites. The results are presented as a heatmap (**Figure 4E**). Specifically, *Proteus mirabilis*, *Enterococcus faecalis*, *Escherichia coli*, and *Clostridium perfringens* exhibited positive correlations with most primary bile acids and negative correlations with secondary bile acids. In contrast, species such as *Akkermansia muciniphila*, *Corynebacterium stationis*, *Desulfovibrio fairfieldensis*, *Alistipes inops*, *Acutalibacter muris*, and *Corynebacterium urealyticum* were positively correlated with secondary bile acids and negatively correlated with most primary bile acids.

**Figure 4:**
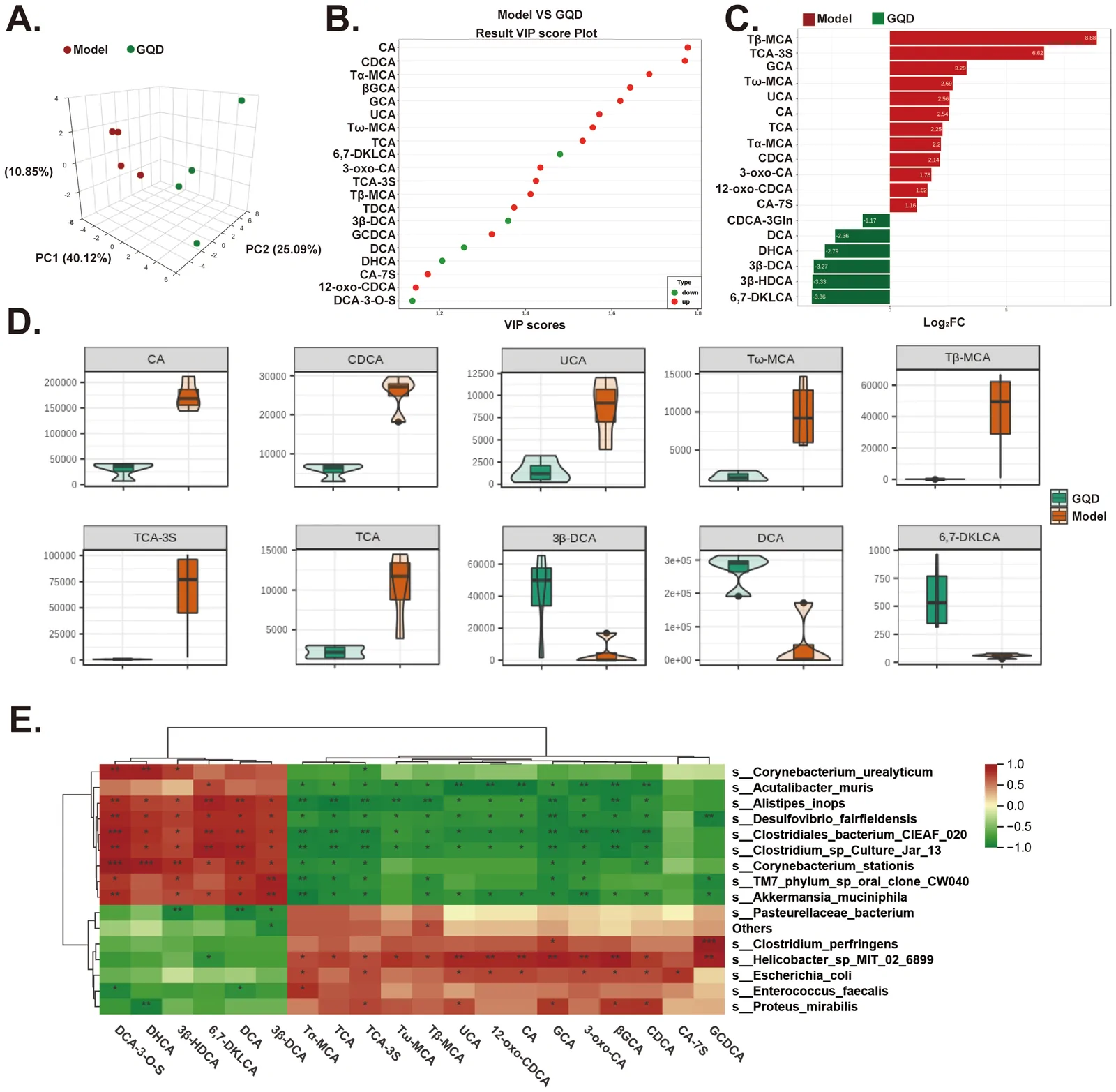
GQD Influences bile acid metabolism in the DSS-induced UC mouse model. **(A)** Principal Component Analysis (PCA) score plot of metabolites; **(B)** Top 20 metabolites with the highest Variable Importance in Projection (VIP) scores in the OPLS-DA model; **(C)** Bar plot of the top 18 differential metabolites based on fold change; **(D)** Violin plots illustrating the overall distribution of differential metabolites; **(E)** Spearman correlation heatmap between gut microbiota and bile acid metabolites (n=4).

### 3.5 Transcriptomic analysis of the therapeutic mechanism of GQD in DSS-induced UC mice

To elucidate the therapeutic mechanism of GQD in UC, RNA sequencing (RNA-seq) was performed on colonic tissues collected from the GQD and Model groups. A total of 486 differentially expressed genes were identified, including 224 upregulated and 262 downregulated genes (**Figure 5A**). Principal Component Analysis (PCA) revealed a distinct separation between the transcriptomic profiles of the two groups (**Figure 5B**). Gene Set Enrichment Analysis (GSEA) identified the top 20 differentially regulated pathways, among which the NOD-like receptor signaling pathway was significantly downregulated (**Figure 5C**). This finding is particularly noteworthy, as the activation of the NLRP3 inflammasome has been reported to trigger pyroptosis, serving as a critical biomarker in the pathogenesis of UC (19). Detailed GSEA of the NOD-like receptor signaling pathway demonstrated a significant negative enrichment (ES = -0.4478, *P* = 9.024e-04). Key molecules within the NLRP3 inflammasome pathway, including IL-18, GSDMD, NLRP3, and IL-1β, exhibited downregulated expression (**Figure 5D**).

**Figure 5:**
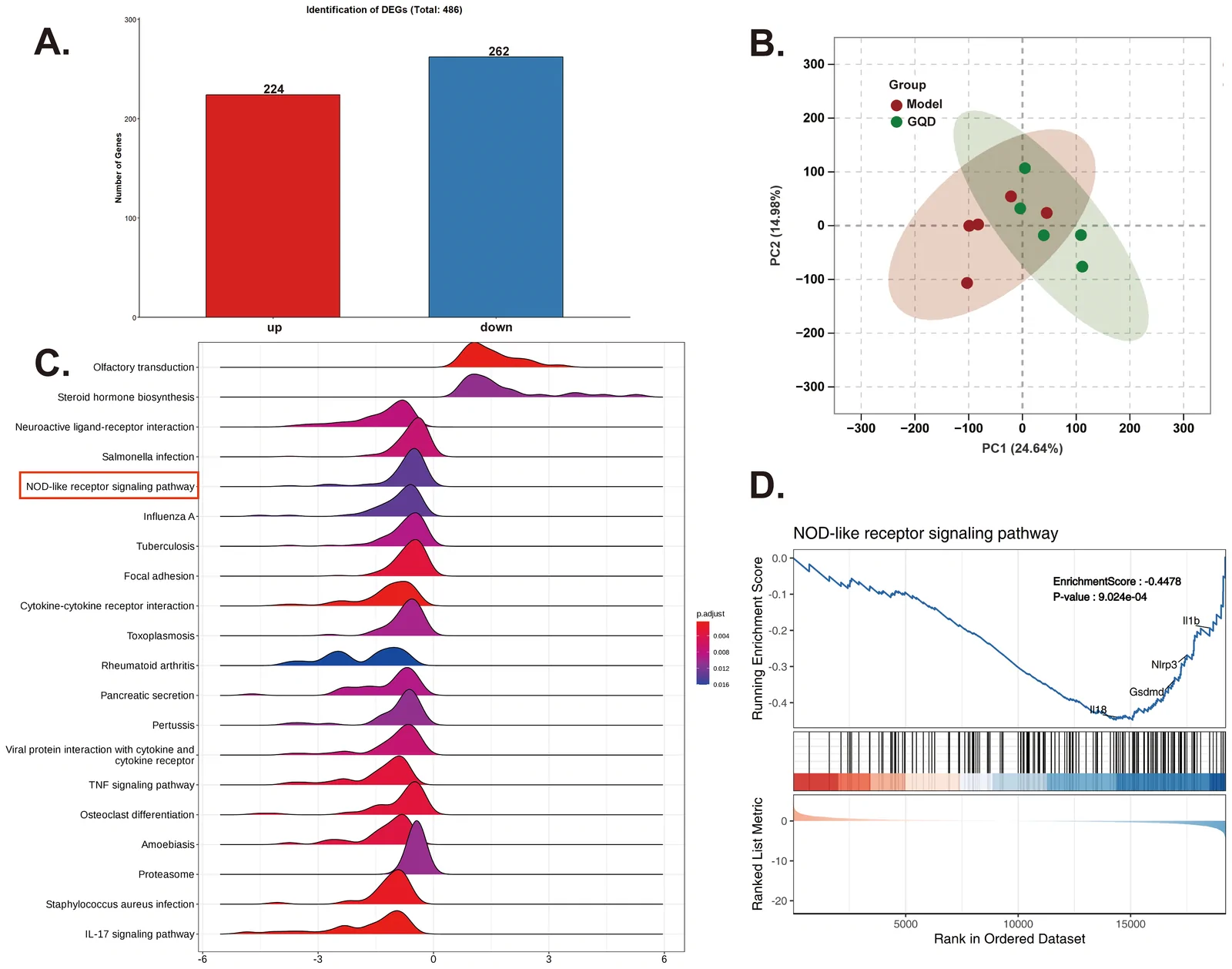
Transcriptomic analysis of the therapeutic mechanism of GQD in DSS-induced UC mice. **(A)** Bar plot showing the number of differentially expressed genes (DEGs); **(B)** Principal Component Analysis (PCA) score plot of the transcriptome; **(C)** Gene Set Enrichment Analysis (GSEA) of the top 20 KEGG pathways; **(D)** GSEA of the NOD-like receptor signaling pathway (n = 5).

### 3.6 GQD attenuates intestinal inflammation in UC mice by inhibiting NLRP3 inflammasome activation

Based on the aforementioned findings, we further performed Western blotting to detect the expression of NLRP3-related proteins in mouse colonic tissues. The levels of associated inflammatory cytokines in colonic tissues were also measured via ELISA. The results showed that, compared with the Model group, GQD-H treatment significantly decreased the expression levels of the NLRP3 protein (P < 0.01) (**Figures 6A, C**). Additionally, other key components of the NLRP3 inflammasome, including pro-Caspase-1, Cleaved-Caspase-1, ASC, and GSDMD-N, were upregulated in the Model group, whereas GQD-H intervention inhibited their expression (*P* < 0.01) (**Figures 6A, E–I**). Furthermore, the expression of intestinal tight junction proteins ZO-1 and Occludin was significantly downregulated in the Model group; however, GQD-H treatment reversed this trend and upregulated the levels of both proteins (*P* < 0.01) (**Figures 6A, B, D**). ELISA results indicated that the levels of TNF-α and IL-18 in colonic tissues were significantly elevated in the Model group, but were markedly reduced following GQD treatment (*P* < 0.01) (**Figures 6J, K**). These findings suggest that GQD alleviates the intestinal inflammatory response in UC mice by inhibiting NLRP3 activation.

**Figure 6:**
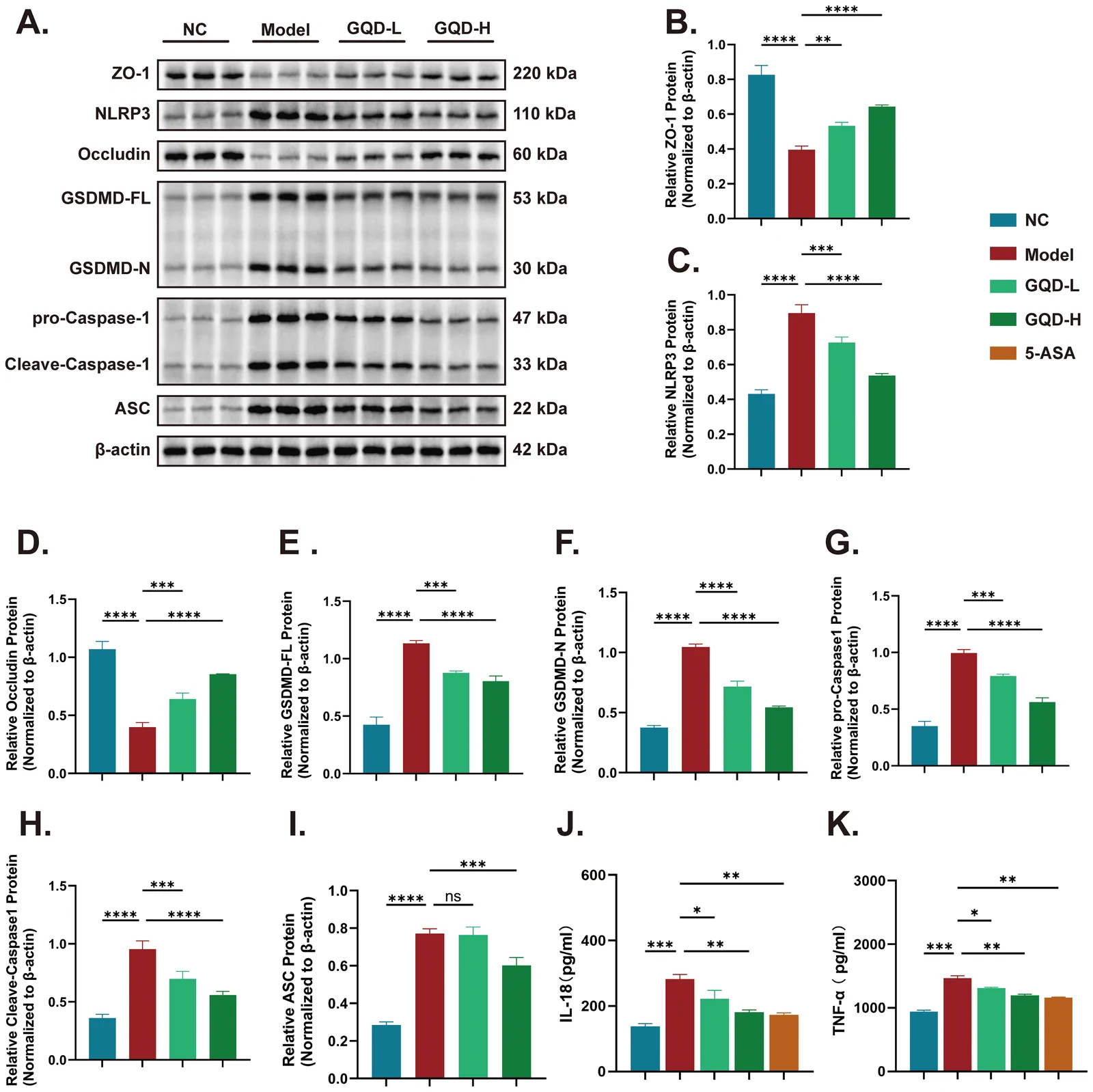
GQD attenuates intestinal inflammation in UC mice by inhibiting NLRP3 inflammasome activation. **(A)** Representative Western blot bands of inflammasome-related proteins and tight junction proteins; **(B–I)** Quantitative analysis of ZO-1, NLRP3, Occludin, GSDMD-FL, GSDMD-N, pro-Caspase-1, Cleaved-Caspase-1 and ASC; **(J)** IL-18 levels in colonic homogenates; **(K)** TNF-α levels in colonic homogenates (ns, not significant; *P < 0.05; **P < 0.01; ***P < 0.001; ****P < 0.0001, n=3).

### 3.7 *In vitro* evaluation of DCA-mediated inhibition of inflammasome activation in Caco-2 cells

First, we performed molecular docking screening of the differential bile acids between the Model and GQD intervention groups. Using the H-bond-first prioritization scheme, DCA emerged as the leading NLRP3-interacting secondary bile acid (**Supplementary S1A, B**). DCA formed 5 hydrogen bonds according to both the Baker-Hubbard and BINANA-like algorithms, while its lowest Vina affinity was -8.141 kcal/mol. Thus, although DCA was not the ligand with the most negative Vina score, it showed the strongest and most consistent hydrogen-bonding profile.

These findings support DCA as the most prominent GQD-associated candidate when hydrogen-bond formation is prioritized; therefore, DCA was selected for subsequent experimental validation. (**Figure 7A**).

**Figure 7:**
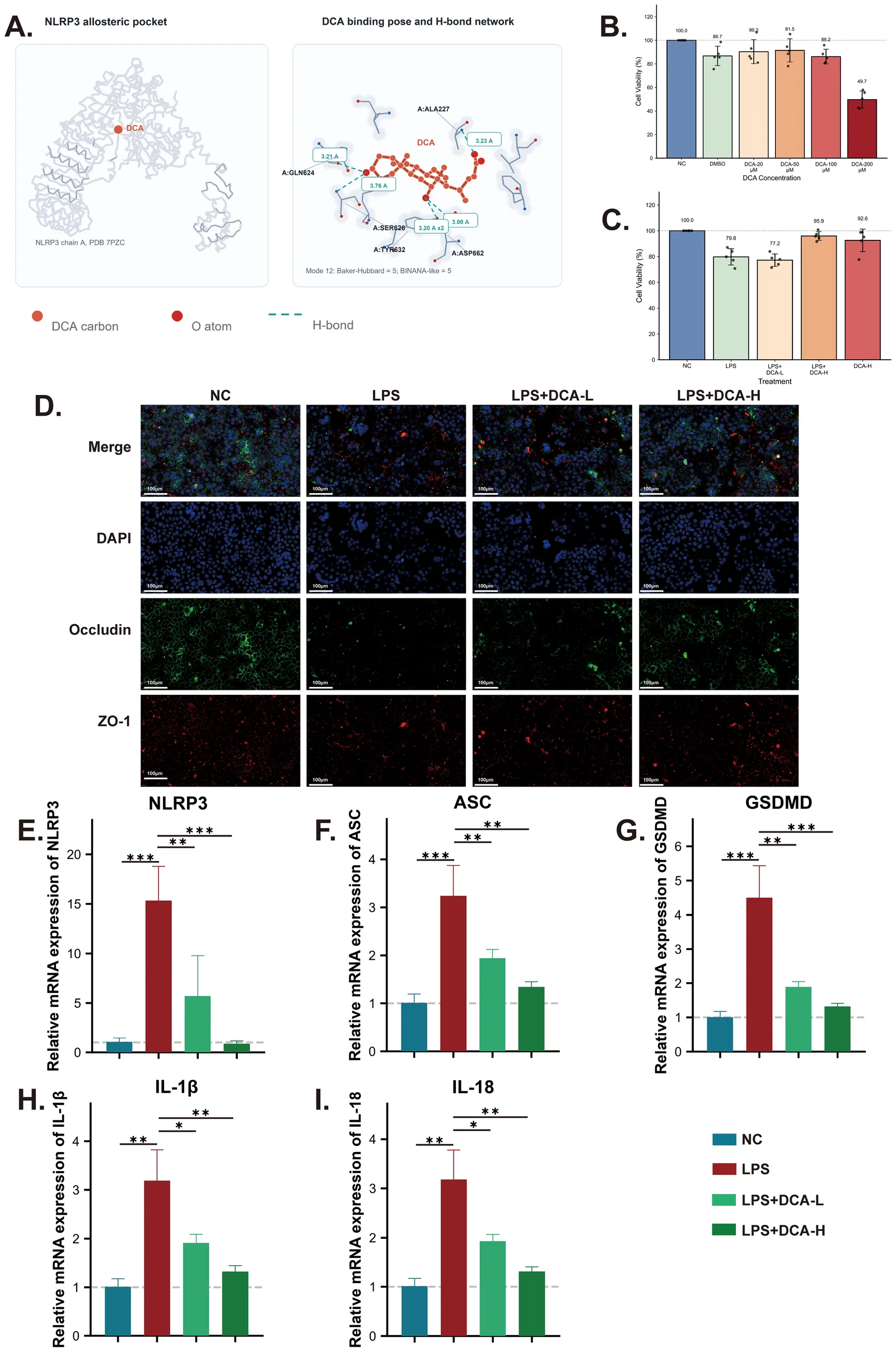
*In vitro* evaluation of DCA-mediated inhibition of inflammasome activation in Caco-2 cells. **(A)** Molecular docking-predicted interaction of deoxycholic acid with the NLRP3 allosteric pocket; **(B)** Effects of different concentrations of DCA on cell viability; **(C)** Effects of LPS and DCA cotreatment on cell viability; **(D)** immunofluorescence staining of ZO-1 and Occludin in cells; **(E–I)** mRNA expression levels of NLRP3, ASC, GSDMD, IL1β, and IL18 in cells from each group (ns, not significant; *P < 0.05; **P < 0.01; ***P < 0.001; ****P < 0.0001, n=3).

A concentration-gradient cytotoxicity assay was first performed to determine the appropriate concentrations of DCA (**Figure 7B**). Based on the results, 20 μM DCA was selected as the low dose (DCA-L), whereas 50 μM DCA was selected as the high dose (DCA-H). Following LPS exposure, DCA treatment attenuated the LPS-induced reduction in Caco-2 cell viability, with a more pronounced protective effect observed at 50 μM than at 20 μM (**Figure 7C**). Cell viability remained above 90% in the LPS + DCA-H group, while 50 μM DCA alone did not appreciably affect cell viability. These findings suggest that DCA protects Caco-2 cells against LPS-induced injury and loss of viability within the tested concentration range.

Immunofluorescence staining of intestinal barrier-associated proteins showed that the fluorescence signals for the tight-junction proteins ZO-1 and Occludin were reduced following LPS treatment, whereas DCA treatment increased these signals (**Figure 7D**).

To investigate whether DCA could suppress LPS-induced activation of the NLRP3 inflammasome, the mRNA expression levels of five key genes—NLRP3, ASC, GSDMD, IL18, and IL1β—were measured using RT-qPCR (**Figures 7E–I**). After stimulation with LPS (10 μg/mL) for 24 h, the mRNA levels of IL18, IL1B, GSDMD, and ASC were significantly higher than those in the NC group (all P < 0.01). These transcriptional changes were consistent with activation of NLRP3 inflammasome-related signaling in Caco-2 cells. Compared with the LPS-only group, the LPS + DCA low-dose group (LPS + DCA-L) showed significantly lower mRNA levels of IL-18, IL-1β, GSDMD, and ASC (all *P* < 0.05). More pronounced reductions were observed in the LPS + DCA high-dose group (LPS + DCA-H). Overall, the expression profiles suggested a dose-related tendency for DCA to suppress the mRNA expression of NLRP3, ASC, GSDMD, IL-18, and IL-1β. The mRNA expression levels of all NLRP3 inflammasome-related genes showed a consistent overall trend.

Taken together, these findings suggest that DCA may attenuate LPS-induced inflammatory responses and help preserve tight-junction-associated proteins in the Caco-2 cell model.

### 3.8 The gut microbiota mediates the inhibitory effect of GQD on NLRP3 inflammasome activation

To verify whether the gut microbiota plays a key mediating role in GQD-induced inhibition of NLRP3 activation, a fecal microbiota transplantation (FMT) experiment was conducted (**Figure 8A**). To assess the engraftment of the transplanted gut microbiota, the relative abundances of four representative bacterial taxa in the feces of recipient mice were quantified using RT-qPCR (**Figures 8B–E**). Compared with the FMT-Model group, the FMT-GQD group exhibited significantly higher relative abundances of *Akkermansia muciniphila* and *Lactobacillus johnsonii* (*P* < 0.05) and significantly lower relative abundances of *Escherichia coli* and *Enterococcus faecalis* (*P* < 0.05). The abundances of all four bacterial taxa differed significantly between the two groups. This trend was consistent with the 16S rRNA gene sequencing results, indicating successful engraftment of the microbiota from GQD-treated donor mice in the intestinal tract of recipient mice. Compared to mice receiving the microbiota from the Model group (FMT-Model), recipient mice transplanted with the GQD-group microbiota (FMT-GQD) exhibited improved intestinal barrier function and attenuated inflammation levels. Western blot analysis revealed that FMT-GQD significantly reduced the expression of NLRP3 and its downstream proteins, including pro-Caspase-1, Cleaved-Caspase-1, ASC, GSDMD-FL, and GSDMD-N (*P* < 0.01) (**Figures 8F, H, J–N**). Concurrently, the expression of tight junction proteins ZO-1 and Occludin was significantly increased (*P* < 0.01) (**Figures 8F, G, I**). ELISA testing further demonstrated that the levels of inflammatory cytokines TNF-α and IL-18 in colonic tissues were significantly lower in the FMT-GQD group (*P* < 0.01) (**Figures 8O, P**). These results confirm that the gut microbiota is a pivotal mediator through which GQD exerts its anti-inflammatory effects.

**Figure 8:**
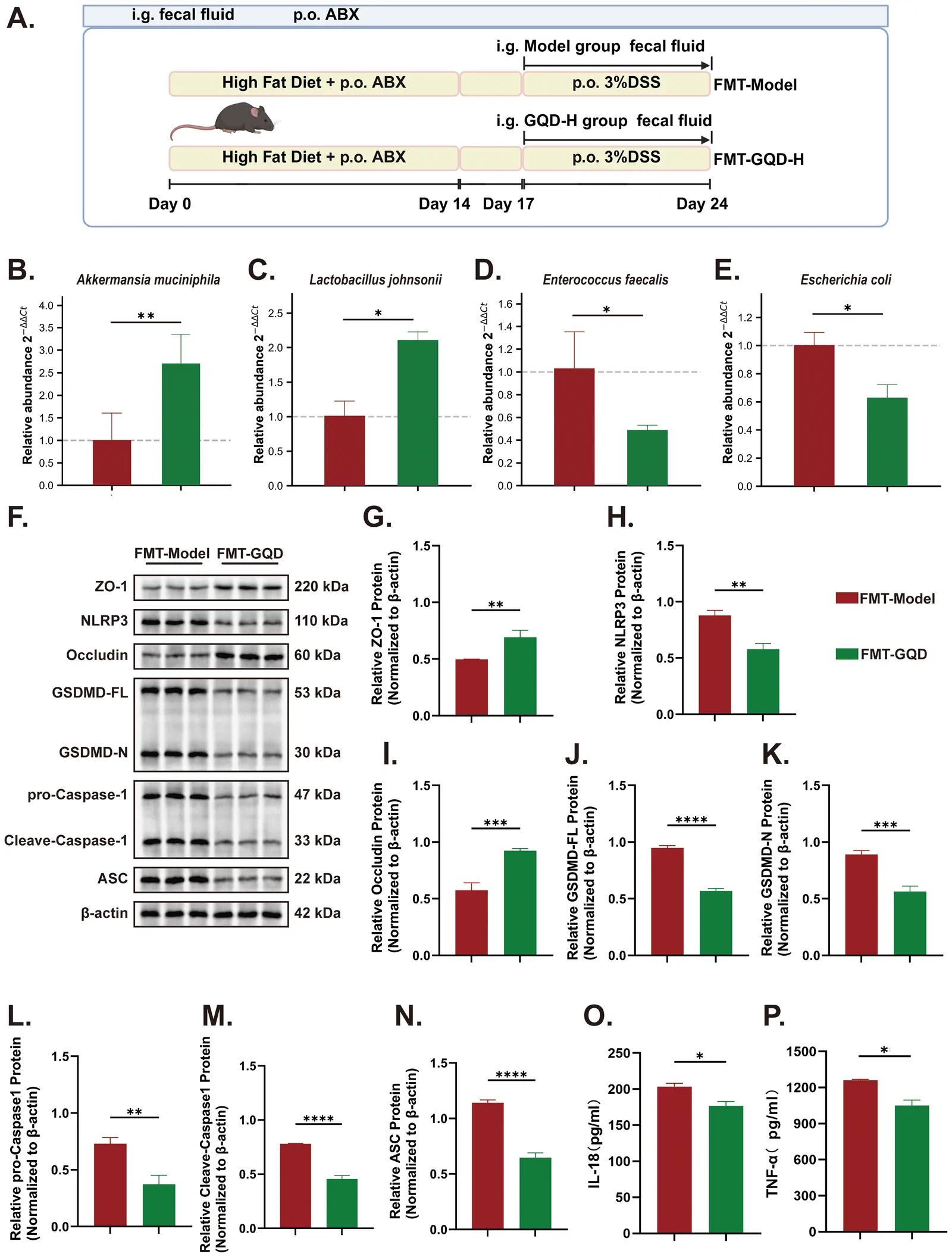
The gut microbiota mediates the inhibitory effect of GQD on NLRP3 inflammasome activation. **(A)** Schematic diagram of Experiment II; **(B–E)** qPCR validation of the engraftment of representative gut bacterial taxa in recipient mice following fecal microbiota transplantation; **(F)** Representative Western blot bands of inflammasome-related proteins and tight junction proteins; **(G–N)** Quantitative analysis of ZO-1, NLRP3, Occludin, GSDMD-FL, GSDMD-N, pro-Caspase-1, Cleaved-Caspase-1 and ASC; **(O)** IL-18 levels in colonic homogenates; **(P)** TNF-α levels in colonic homogenates (ns, not significant; *P < 0.05; **P < 0.01; ***P < 0.001; ****P < 0.0001; antibiotic cocktail, ABX; fecal microbiota transplantation, FMT; oral gavage, i.g.; oral administration, p.o.; GQD high-dose group, GQD-H, n=3).

## 4 Discussion

UC is a chronic inflammatory bowel disease characterized by a complex pathogenesis. Accumulating clinical and experimental evidence indicates that an HFD is a key environmental factor driving the exacerbation of UC pathological progression and the protraction of symptoms (20, 21). Gegen Qinlian Decoction was first recorded in the *Shanghan Lun* (Treatise on Febrile Diseases) for treating “Xie Li” (dysentery-like syndromes) with symptoms including diarrhea, tenesmus, and fever. These manifestations closely resemble the clinical presentation of active ulcerative colitis. Recent clinical trials have shown that GQD reduces the Ulcerative Colitis Endoscopic Index of Severity (UCEIS) and maintains a low recurrence rate in UC patients (22).

The mechanisms by which GQD ameliorates UC—especially under the exacerbating influence of an HFD—remain incompletely understood. This study provides multi-omics evidence that GQD alleviates UC in an HFD-preconditioned DSS model by remodeling the gut microbiota–bile acid (BA) axis and inhibiting NLRP3 inflammasome-mediated pyroptosis. Our findings connect the traditional use of GQD for intestinal inflammatory disorders to modern insights into the “microbiota-metabolite-immune” regulatory network.

First, the chemical constituents of GQD were qualitatively analyzed using UPLC-MS/MS, identifying major active components such as berberine and baicalin. A substantial body of evidence has demonstrated that these active constituents can significantly ameliorate ulcerative colitis or exert anti-inflammatory effects. Berberine has been reported to markedly restore goblet cell numbers and upregulate tight junction protein and mucin expression, thereby alleviating DSS-induced colitis (23). Baicalin suppresses inflammation by inhibiting CD14 expression and the MyD88/NF-κB p65 pathway in HT-29 cells (24), and has also been shown to reduce the secretion of pro-inflammatory cytokines, including IL-1β, IL-18, and TNF-α, downregulate ROS levels, and inhibit ERK phosphorylation, thereby suppressing NLRP3-mediated pyroptosis (25). Coptisine and palmatine significantly activate the aryl hydrocarbon receptor (AhR) and enhance the activity of its downstream gene CYP1A1, while berberine can also activate AhR by modulating the gut microbiota and its tryptophan metabolites, ultimately restoring impaired intestinal barrier function (26). Notably, these compounds exhibit low intestinal bioavailability and poor systemic absorption following oral administration. They often require microbial biotransformation within the gut to enhance their absorption or bioactivity, thereby fully exerting their therapeutic effects. For instance, while the oral bioavailability of berberine is less than 1%, it can be metabolized by the gut microbiota into dihydroberberine, which possesses superior pharmacological activity. This metabolite exerts potent anti-inflammatory effects by maintaining intestinal barrier integrity and inhibiting the TLR4-MyD88-NF-κB signaling pathway (27, 28). Additionally, flavonoids such as baicalin and wogonoside undergo hydrolysis by microbial β-glucuronidase in the gut. They are converted into more lipophilic aglycone forms—baicalein and wogonin—before they can be effectively absorbed (29); studies have shown that these transformed active components target and inhibit NLRP3 inflammasome activation in intestinal epithelial cells, thereby alleviating colonic mucosal damage (30). Similarly, glycyrrhizinate and liquiritin require microbial deglycosylation to be converted into glycyrrhetic acid and liquiritigenin, these metabolites repair DSS-induced intestinal barrier impairment by upregulating the expression of tight junction proteins such as ZO-1 and Occludin (31). The aforementioned evidence suggests that the efficacy of GQD largely depends on its ability to reshape the intestinal microecology, which in turn facilitates the full manifestation of its medicinal utility.

Clinical studies have demonstrated that gut microbiota dysbiosis is not only a key pathological feature of ulcerative colitis but also a core factor driving its progression (32). By expressing BSH, the gut microbiota can directly influence the conjugation status of taurine or glycine with bile acids, thereby modulating the composition of bile acids. In addition, the microbiota further generates secondary bile acids via 7α-HSDH and the 7α-dehydroxylation of deconjugated bile acids (33). Previous studies have indicated that intestinal bacteria harboring the BSH gene are mainly concentrated at the genus level in *Bacteroides*, *Clostridium*, *Bifidobacterium*, and *Lactobacillus* (34). In the present study, we found that following GQD intervention, the relative abundances of *Bacteroides* and *Lactobacillus* were significantly increased. More importantly, we observed that GQD not only modulates the gut microbiota structure in UC, but also promotes the growth of beneficial bacteria while inhibiting the proliferation of harmful bacteria, thereby achieving an optimization of the intestinal microecological environment at a deeper level. In this study, we similarly observed characteristic gut microecological alterations in the HFD-preconditioned DSS model, marked by the depletion of certain probiotics and the enrichment of pathogenic bacteria. Our findings indicate that GQD intervention can specifically modulate the gut microbiota structure, effectively reversing this pathological microbial shift. GQD significantly enriched beneficial bacteria such as *Akkermansia muciniphila* and *Lactobacillus murinus*, while inhibiting the proliferation of opportunistic pathogens like *Clostridium perfringens*. As a representative intestinal probiotic, *A. muciniphila* effectively promotes mucus secretion from goblet cells, regulates the expression of tight junction proteins, and accelerates intestinal epithelial regeneration, thereby strengthening barrier function. The roles of *Lactobacillus murinus* in modulating immunity, improving metabolism, and protecting the intestinal barrier have been validated across multiple animal studies. The opportunistic pathogen *Clostridium perfringens* typically undergoes overproliferation under specific conditions such as gut dysbiosis or compromised immunity, producing toxins that lead to enteritis. The gut microecology reshaped by GQD creates a favorable environment for promoting intestinal immune-metabolic homeostasis.

As critical signaling molecules of microbial metabolism, bile acids (BAs) serve as a pivotal link between the microecology and host immune homeostasis. Clinical evidence indicates that patients with UC often exhibit significant dysregulation of the bile acid profile, characterized by decreased levels of secondary BAs (e.g., DCA and LCA) and elevated levels of primary BAs (e.g., CA) (35). High concentrations of primary BAs, such as CA and CDCA, act as potent endogenous pro-inflammatory stimuli that can induce oxidative stress and trigger immune responses (36). Conversely, secondary BAs mediate anti-inflammatory effects and alleviate symptoms of colitis (37, 38). Consistently, this study observed an aberrant accumulation of primary BAs—represented by CA, CDCA, and TCA—in the colons of UC mice. This was accompanied by a metabolic shift characterized by a depletion of secondary BAs, such as DCA, 6,7-DKLCA and 3β-DCA. GQD intervention significantly ameliorated this metabolic dysregulation, effectively reducing the pathological concentrations of pro-inflammatory primary BAs and elevating secondary BA levels. This reshaping of the bile acid profile synergistically induced a transition in the intestinal immune microenvironment toward an “anti-inflammatory” phenotype. These findings reveal the role of GQD in modulating immuno-metabolic homeostasis, providing metabolomic evidence for its clinical efficacy in alleviating intestinal inflammation.

In this study, molecular docking was performed using the NLRP3 structure as the receptor. The candidate metabolites included in the docking analysis were not selected from the complete set of differential metabolites but were restricted to the secondary bile acids and secondary bile acid derivatives identified by the LEfSe analysis. This inclusion strategy was based on the preceding evidence that GQD intervention was associated with alterations in gut microbial composition. Accordingly, priority was given to secondary bile acid–related metabolites whose biotransformation or regulation may involve the gut microbiota, thereby strengthening the internal consistency of the proposed “gut microbiota–bile acid metabolism–NLRP3” mechanistic axis. DCA was ultimately prioritized as a candidate metabolite linking GQD-associated bile acid remodeling to the regulation of intestinal epithelial inflammation. Although DCA did not yield the most negative Vina score, both the Baker–Hubbard and BINANA-like algorithms identified five hydrogen bonds, with ALA227, GLN624, and TYR632 recurring in both analyses. These findings suggest cross-algorithm consistency in the predicted binding pose and identify candidate residues for subsequent site-directed mutagenesis. Previous studies have similarly emphasized that the reliability of docking scores is constrained by conformational sampling and scoring functions and that docking predictions require validation through biophysical and functional experiments (39).

Within the concentration range that did not produce appreciable cytotoxicity, 20-50 μM DCA reduced the LPS-induced mRNA expression of NLRP3, ASC, GSDMD, IL18, and IL1B. All five NLRP3 inflammasome-related genes exhibited a consistent dose-related pattern, with more pronounced reductions observed at the higher DCA concentration. A statistically significant dose-related trend was also observed for NLRP3, suggesting that the effect of DCA was not restricted to downstream inflammatory mediators but also involved the transcriptional regulation of NLRP3 itself. These findings suggest that DCA may attenuate LPS-induced priming of the NLRP3 inflammasome pathway at the transcriptional level. Previous studies have shown that bile acids can promote the phosphorylation and ubiquitination of NLRP3 through the TGR5–cAMP–PKA axis, thereby suppressing inflammasome activation (40). Other studies have also reported that low concentrations of DCA exert anti-inflammatory effects in epithelial cell–related inflammatory models through the TGR5–cAMP–PKA–NF-κB/NLRP3 pathway (41).

Previous studies have established that high concentrations of primary bile acids can drive the activation of the NLRP3 inflammasome, thereby exacerbating pathological impairment of the intestinal barrier (42). The hyperactivation of the NLRP3 inflammasome is considered a central event driving the “inflammatory storm” in ulcerative colitis (43). Our transcriptomic sequencing and Western blot analyses further validated that GQD significantly downregulates the expression of NLRP3, ASC, and Caspase-1. This downregulation effectively blocks the pyroptosis process mediated by the N-terminal fragment of GSDMD-N. Colonic IL-18 and TNF-α were also reduced, indicating that GQD intercepts the pyroptotic program at an early stage. Pyroptosis, a pro-inflammatory form of programmed cell death executed by GSDMD, is a key driver of mucosal damage in UC. Excessive primary BAs can act as damage-associated molecular patterns (DAMPs) that activate the NLRP3 inflammasome, triggering caspase-1-dependent pyroptosis and the release of pro-inflammatory cytokines. By remodeling the BA profile to reduce primary BA accumulation and elevate secondary BA levels, GQD indirectly suppresses this inflammasome cascade. The upregulation of tight junction proteins ZO-1 and occludin further confirms the restoration of intestinal barrier integrity.

To establish causality, we performed fecal microbiota transplantation (FMT) from GQD-treated donors into antibiotic-pretreated recipient mice. FMT-GQD recipients recapitulated the key protective phenotypes observed with direct GQD administration: reduced NLRP3 inflammasome activation, lower GSDMD-N expression, restored ZO-1/occludin levels, and decreased IL-18 and TNF-α. These results demonstrate that the gut microbiota is a necessary mediator—not merely a bystander—of GQD’s anti-inflammatory and barrier-protective effects. This FMT finding is particularly significant from an ethnopharmacological perspective. It supports the long-held concept in TCM that orally administered herbal formulas exert their therapeutic actions, at least in part, through modulation of the intestinal environment. The fact that microbiota from GQD-treated mice can confer protection even without direct GQD administration highlights the central role of the gut ecosystem in translating GQD’s pharmacological potential into clinical benefit. In summary, GQD orchestrates the remodeling of the gut microbiota-mediated bile acid metabolic profile. This metabolic shift subsequently intercepts the NLRP3/GSDMD-mediated pyroptosis program, thereby exerting potent anti-inflammatory effects and conferring significant protection to the intestinal barrier.

Several limitations of this study should be acknowledged when interpreting these findings. The sample sizes for 16S rRNA sequencing and targeted bile acid metabolomics were relatively modest (n = 4 per group), and the causal relationship between bile acids and NLRP3 was evaluated using only one representative secondary bile acid (DCA) in a single intestinal epithelial cell line. In addition, the FMT experiments were conducted in antibiotic-treated pseudo-germ-free mice rather than germ-free animals, and the bioactivity of certain GQD constituents, such as berberine and baicalin, may depend on gut microbial metabolism. Therefore, the proposed regulatory cascade should be regarded as a plausible mechanism supported under the current experimental conditions rather than a universally established model. Nevertheless, the consistency across multi-omics, FMT, and biochemical analyses provides strong support for the central findings, and the above limitations do not compromise the main conclusions of this study.

## 5 Conclusion

In summary, this study demonstrates that GQD alleviates UC induced by combined HFD and DSS exposure by remodeling the gut microbiota and correcting BA metabolic dysregulation, which in turn suppresses NLRP3 inflammasome-mediated pyroptosis and preserves intestinal barrier integrity. Our findings mechanistically link the traditional use of GQD for intestinal disorders to the modern understanding of the microbiota-BA-NLRP3 axis. The gut microbiota-BA axis emerges as a critical therapeutic target for UC, and this study provides robust experimental support for the clinical application of GQD in UC patients, particularly those with a high-fat diet background.

## Data Availability

The original contributions presented in the study are included in the article/**Supplementary Material**. Further inquiries can be directed to the corresponding authors.

## References

[1] Rubin DT Ananthakrishnan AN Siegel CA Barnes EL Long MD . ACG clinical guideline update: Ulcerative colitis in adults. (2025) 120:. doi: 10.14309/ajg.0000000000003463 40701556

[2] Ng SC Shi HY Hamidi N Underwood FE Tang W Benchimol EI . Worldwide incidence and prevalence of inflammatory bowel disease in the 21st century: A systematic review of population-based studies. Lancet. (2017) 390:2769–78. doi: 10.1016/S0140-6736(17)32448-0 29050646

[3] Cui G Yuan A . A systematic review of epidemiology and risk factors associated with Chinese inflammatory bowel disease. Front Med. (2018) 5:183. doi: 10.3389/fmed.2018.00183 29971235 PMC6018203

[4] Gao J Li H Wang Z Jiang G Pan L Jiang Z . Cirsiliol alleviates experimental inflammatory bowel disease via restoration of intestinal barrier integrity and inhibition of NF-κB and MAPK pathways. Sci Rep. (2025) 15:24408. doi: 10.1038/s41598-025-09896-x 40629068 PMC12238409

[5] Singh S Dulai PS Zarrinpar A Ramamoorthy S Sandborn WJ . Obesity in IBD: Epidemiology, pathogenesis, disease course and treatment outcomes. Nat Rev Gastroenterol Hepatol. (2016) 14:110–21. doi: 10.1038/nrgastro.2016.181 27899815 PMC5550405

[6] Zhou Y Wang B Wang Q Wang F Mou X Gong L . *Lactobacillus plantarum* Lac16 alleviates dextran sodium sulfate-induced colitis in mice by suppressing NLRP3 inflammasome overactivation through microbiota-derived isobutyric acid. mBio. (2025) 16:e02392-25. doi: 10.1128/mbio.02392-25 41171006 PMC12691655

[7] He H Guo X Xu M Tan Z Tan C Li X . High-fat diet exacerbates experimental colitis by inhibiting lysosomal function via the STAT3-TFEB axis. Autophagy. (2026), 1–24. doi: 10.1080/15548627.2026.2678426 42165414 PMC13618857

[8] Kriaa A Mariaule V Jablaoui A Rhimi S Mkaouar H Hernandez J . Bile acids: Key players in inflammatory bowel diseases? Cells. (2022) 11:901. doi: 10.3390/cells11050901 35269523 PMC8909766

[9] Sommersberger S Gunawan S Elger T Fererberger T Loibl J Huss M . Altered fecal bile acid composition in active ulcerative colitis. Lipids Health Dis. (2023) 22:199. doi: 10.1186/s12944-023-01971-4 37980492 PMC10656844

[10] Yang Z-H Liu F Zhu X-R Suo F-Y Jia Z Yao S-K . Altered profiles of fecal bile acids correlate with gut microbiota and inflammatory responses in patients with ulcerative colitis. World J Gastroenterol. (2021) 27:3609–29. doi: 10.3748/wjg.v27.i24.3609 34239273 PMC8240054

[11] Zheng M Zhai Y Yu Y Shen J Chu S Focaccia E . TNF compromises intestinal bile-acid tolerance dictating colitis progression and limited infliximab response. Cell Metab. (2024) 36:2086–2103.e9. doi: 10.1016/j.cmet.2024.06.008 38971153

[12] Sokolova M Yang K Hansen SH Louwe MC Kummen M Hov JER . NLRP3 inflammasome deficiency attenuates metabolic disturbances involving alterations in the gut microbial profile in mice exposed to high fat diet. Sci Rep. (2020) 10:21006. doi: 10.1038/s41598-020-76497-1 33273482 PMC7712828

[13] Xu B-L Zhang G-J Ji Y-B . Active components alignment of gegenqinlian decoction protects ulcerative colitis by attenuating inflammatory and oxidative stress. J Ethnopharmacol. (2015) 162:253–60. doi: 10.1016/j.jep.2014.12.042 25557032

[14] Huang J Zhang J Wang Y Ma J Yang X Guo X . Scientific evidence of Chinese herbal medicine (Gegen Qinlian decoction) in the treatment of ulcerative colitis. Gastroenterol Res Pract. (2022) 2022:1–8. doi: 10.1155/2022/7942845 35356743 PMC8958105

[15] Mu S Zheng Z-J Lu J-Z Pan L-Y Ma B-L . Natural nanoparticles in Gegen–Qinlian decoction promote the colonic absorption of active constituents in mice with dextran sulfate sodium-induced ulcerative colitis. Pharmaceuticals. (2025) 18:1718. doi: 10.3390/ph18111718 41304962 PMC12654978

[16] Deng L Shi Y Liu P Wu S Lv Y Xu H . GeGen QinLian decoction alleviate influenza virus infectious pneumonia through intestinal flora. BioMed Pharmacother. (2021) 141:111896. doi: 10.1016/j.biopha.2021.111896 34246956

[17] Liu J Liu Y Huang C He C Yang T Ren R . Quercetin‐driven *Akkermansia muciniphila* alleviates obesity by modulating bile acid metabolism via an ILA/m^6^ A/CYP8B1 signaling. Adv Sci. (2025) 12:2412865. doi: 10.1002/advs.202412865 39888270 PMC11948036

[18] Lin Z Feng Y Wang J Men Z Ma X . Microbiota governs host chenodeoxycholic acid glucuronidation to ameliorate bile acid disorder induced diarrhea. Microbiome. (2025) 13:36. doi: 10.1186/s40168-024-02011-8 39905483 PMC11792533

[19] Cruz H Shah P Wohlgemuth N Ketchum R Nassif I Hu C-A . Pyroptosis in ulcerative colitis: Biomarkers and therapeutic targets. J BioMed Sci. (2025) 32:106. doi: 10.1186/s12929-025-01206-x 41340115 PMC12673710

[20] Hashash JG Elkins J Lewis JD Binion DG . AGA clinical practice update on diet and nutritional therapies in patients with inflammatory bowel disease: Expert review. Gastroenterology. (2024) 166:521–32. doi: 10.1053/j.gastro.2023.11.303 38276922

[21] Fritsch J Garces L Quintero MA Pignac-Kobinger J Santander AM Fernández I . Low-fat, high-fiber diet reduces markers of inflammation and dysbiosis and improves quality of life in patients with ulcerative colitis. Clin Gastroenterol Hepatol. (2021) 19:1189–1199.e30. doi: 10.1016/j.cgh.2020.05.026 32445952

[22] Chen Y Li R Yuan Y He H Yang W Yuan C . Efficacy and safety of Gegen Qinlian decoction combined with mesalazine for the treatment of UC: A meta-analysis and systematic review. Med (Baltimore). (2025) 104:e42266. doi: 10.1097/MD.0000000000042266 40725879 PMC12303433

[23] Li C Dong N Wu B Mo Z Xie J Lu Q . Dihydroberberine, an isoquinoline alkaloid, exhibits protective effect against dextran sulfate sodium-induced ulcerative colitis in mice. Phytomedicine. (2021) 90:153631. doi: 10.1016/j.phymed.2021.153631 34253428

[24] Fu Y Xu B Huang S Luo X Deng X Luo S . Baicalin prevents LPS-induced activation of TLR4/NF-κB p65 pathway and inflammation in mice via inhibiting the expression of CD14. Acta Pharmacol Sin. (2020) 42:88–96. doi: 10.1038/s41401-020-0411-9 32457419 PMC7921675

[25] Guo M Wu H Zhang J Zhao L He J Yu Z . Baicalin n-butyl ester alleviates inflammatory bowel disease and inhibits pyroptosis through the ROS/ERK/P-ERK/NLRP3 pathway *in vivo* and *in vitro*. BioMed Pharmacother. (2025) 186:118012. doi: 10.1016/j.biopha.2025.118012 40168722

[26] Tan B Zhang J Kang A Zhang L Fang D Wu H . Coptisine activates aryl hydrocarbon receptor to regulate colonic epithelial homeostasis in DSS induced ulcerative colitis and TNF-α challenged intestinal organoids. Phytomedicine. (2025) 145:157054. doi: 10.1016/j.phymed.2025.157054 40639239

[27] Li C Ai G Wang Y Lu Q Luo C Tan L . Oxyberberine, a novel gut microbiota-mediated metabolite of berberine, possesses superior anti-colitis effect: Impact on intestinal epithelial barrier, gut microbiota profile and TLR4-MyD88-NF-κB pathway. Pharmacol Res. (2020) 152:104603. doi: 10.1016/j.phrs.2019.104603 31863867

[28] Feng R Zhao Z-X Ma S-R Guo F Wang Y Jiang J-D . Gut microbiota-regulated pharmacokinetics of berberine and active metabolites in beagle dogs after oral administration. Front Pharmacol. (2018) 9:214. doi: 10.3389/fphar.2018.00214 29618977 PMC5871679

[29] Noh K Kang Y Nepal M Jeong K Oh D Kang M . Role of intestinal microbiota in baicalin-induced drug interaction and its pharmacokinetics. Molecules. (2016) 21:337. doi: 10.3390/molecules21030337 26978333 PMC6273104

[30] Sun Y Zhao Y Yao J Zhao L Wu Z Wang Y . Wogonoside protects against dextran sulfate sodium-induced experimental colitis in mice by inhibiting NF-κB and NLRP3 inflammasome activation. Biochem Pharmacol. (2015) 94:142–54. doi: 10.1016/j.bcp.2015.02.002 25677765

[31] Jeon Y-D Kang S-H Bang K-S Chang Y-N Lee J-H Jin J-S . Glycyrrhetic acid ameliorates dextran sulfate sodium-induced ulcerative colitis *in vivo*. Molecules. (2016) 21:523. doi: 10.3390/molecules21040523 27110761 PMC6273862

[32] Liang Y Li Y Lee C Yu Z Chen C Liang C . Ulcerative colitis: Molecular insights and intervention therapy. Mol BioMed. (2024) 5:42. doi: 10.1186/s43556-024-00207-w 39384730 PMC11464740

[33] Wan Y . Unconjugated and secondary bile acid profiles in response to higher-fat, lower-carbohydrate diet and associated with related gut microbiota: A 6-month randomized controlled-feeding trial. Clin Nutr. (2020) 39:395–404. doi: 10.1016/j.clnu.2019.02.037 30876827

[34] Chiang JYL . Bile acids: Regulation of synthesis. J Lipid Res. (2009) 50:1955–66. doi: 10.1194/jlr.R900010-JLR200 19346330 PMC2739756

[35] Lloyd-Price J Arze C Ananthakrishnan AN Schirmer M Avila-Pacheco J Poon TW . Multi-omics of the gut microbial ecosystem in inflammatory bowel diseases. Nature. (2019) 569:655–62. doi: 10.1038/s41586-019-1237-9 31142855 PMC6650278

[36] Li T Hasan MN Gu L . Bile acids regulation of cellular stress responses in liver physiology and diseases. eGastroenterology. (2024) 2:e100074. doi: 10.1136/egastro-2024-100074 39027418 PMC11257078

[37] Li N Ma P Li Y Shang X Nan X Shi L . Gut microbiota-derived 12-ketolithocholic acid suppresses the IL-17A secretion from colonic group 3 innate lymphoid cells to prevent the acute exacerbation of ulcerative colitis. Gut Microbes. (2023) 15:2290315. doi: 10.1080/19490976.2023.2290315 38062857 PMC10730201

[38] Han B Wen H Li Y Wang Y Lv X Kang M . Gut microbial production of lithocholic acid reprograms pro-resolutive macrophages to enhance vedolizumab responsiveness via the TGR5/FXR–NF-κB axis. ISME J. (2026) 20:wrag028. doi: 10.1093/ismejo/wrag028 41697021 PMC12998432

[39] Pantsar T Poso A . Binding affinity via docking: Fact and fiction. Molecules. (2018) 23:1899. doi: 10.3390/molecules23081899 30061498 PMC6222344

[40] Guo C Xie S Chi Z Zhang J Liu Y Zhang L . Bile acids control inflammation and metabolic disorder through inhibition of NLRP3 inflammasome. Immunity. (2016) 45:802–16. doi: 10.1016/j.immuni.2016.09.008 27692610

[41] Zhao C Wu K Hao H Zhao Y Bao L Qiu M . Gut microbiota-mediated secondary bile acid alleviates Staphylococcus aureus-induced mastitis through the TGR5-cAMP-PKA-NF-κB/NLRP3 pathways in mice. NPJ Biofilms Microbiomes. (2023) 9:8. doi: 10.1038/s41522-023-00374-8 36755021 PMC9908919

[42] Holtmann TM Inzaugarat ME Knorr J Geisler L Schulz M Bieghs V . Bile acids activate NLRP3 inflammasome, promoting murine liver inflammation or fibrosis in a cell type-specific manner. Cells. (2021) 10:2618. doi: 10.3390/cells10102618 34685598 PMC8534222

[43] Wang J Hua S Bao H Yuan J Zhao Y Chen S . Pyroptosis and inflammasomes in cancer and inflammation. MedComm. (2023) 4:e374. doi: 10.1002/mco2.374 37752941 PMC10518439

