# Supplementary material for: Gegen Qinlian decoction alleviates ulcerative colitis by remodeling the gut microbiota-bile acid axis and inhibiting NLRP3-mediated pyroptosis
Source: Front Immunol. 2026 Oct 1;17:1895206. doi: 10.3389/fimmu.2026.1895206 (PMC13669093; doi:10.3389/fimmu.2026.1895206)
Supplement: Supplementary file 1 [file SupplementaryFile1.docx]

**Supplementary Figure 1**

**Figure S1. Comparison of molecular docking results for secondary bile acids against NLRP3.** A) Hydrogen-bond-prioritized docking ranking of secondary bile acids; B) Integrated distribution of hydrogen-bond counts and Vina docking scores for secondary bile acids.

**Supplementary Figure 2**

**Figure S2. 16S rRNA gene alpha diversity.** A) Rank-abundance curves for each group; B) Abundance-based Coverage Estimator (ACE); (C) Chao1 richness estimator; (D) Shannon diversity index; E) Simpson diversity index.
